# The guanine nucleotide exchange factor DOCK5 negatively regulates osteoblast differentiation and BMP2-induced bone regeneration via the MKK3/6 and p38 signaling pathways

**DOI:** 10.1038/s12276-024-01372-2

**Published:** 2025-01-01

**Authors:** Ju Ang Kim, Soomin Im, Jiwon Lim, Jung Min Hong, Hye Jung Ihn, Jong-Sup Bae, Jung-Eun Kim, Yong Chul Bae, Eui Kyun Park

**Affiliations:** 1 Department of Pathology and Regenerative Medicine, School of Dentistry, IHBR, Kyungpook National University, Daegu, 41940 Republic of Korea; 2 Cell and Matrix Research Institute, Kyungpook National University, Daegu, 41944 Republic of Korea; 3 Research Institute of Pharmaceutical Sciences, College of Pharmacy, Kyungpook National University, Daegu, 41566 Republic of Korea; 4 Department of Molecular Medicine, Cell and Matrix Research Institute, School of Medicine, Kyungpook National University, Daegu, 41944 Republic of Korea; 5 Department of Anatomy and Neurobiology, School of Dentistry, Kyungpook National University, Daegu, 41940 Republic of Korea

**Keywords:** Growth factor signalling, Bone development

## Abstract

DOCK5 (dedicator of cytokinesis 5), a guanine nucleotide exchange factor for Rac1, has been implicated in BMP2-mediated osteoblast differentiation, but its specific role in osteogenesis and bone regeneration remained unclear. This study investigated the effect of DOCK5 on bone regeneration using C21, a DOCK5 chemical inhibitor, and *Dock5*-deficient mice. Osteoblast differentiation and bone regeneration were analyzed using bone marrow mesenchymal stem cells (BMSCs) and various animal models. C21 significantly enhanced osteoblast differentiation and mineral deposition in mouse MC3T3-E1 cells and in human and mouse BMSCs. *Dock5* knockout (KO) mice exhibited increased bone mass and mineral apposition rate, with their BMSCs showing enhanced osteoblast differentiation. Calvarial defect and ectopic bone formation models demonstrated significant induction of bone regeneration in *Dock5* KO mice compared to wild-type (WT) mice. Moreover, DOCK5 inhibition by C21 in WT mice enhanced BMP2-induced subcutaneous ectopic bone formation. The mechanism responsible for enhanced bone formation induced by DOCK5 inhibition may involve the suppression of Rac1 under TAK1, accompanied by the activation of MKK3/6 and p38 induced by BMP2. These findings strongly suggest that DOCK5 negatively regulates osteoblast differentiation and bone regeneration through signaling pathways involving TAK1, MKK3/6, and p38, providing new insights into potential therapeutic strategies for bone regeneration.

## Enhanced Bone Regeneration Linked to DOCK5 Deficiency

Bone regeneration involves repairing damaged or lost bone by forming new tissue, supported by growth factors like BMP2 (bone morphogenetic protein 2). However, high doses of BMP2 can cause side effects. In this study, researchers found that blocking DOCK5, a protein involved in cell signaling, enhances BMP2’s ability to promote bone growth. They used chemical inhibitors and genetically modified mice to study DOCK5’s role in bone formation, growing bone cells in vitro and testing bone regeneration in vivo. Results showed that inhibiting DOCK5 increased bone growth and mineralization when BMP2 was present. The study suggests that targeting DOCK5 could enhance BMP2’s effects, allowing for lower doses in bone healing and offering a new approach for bone repair therapies. Future research may explore the use of DOCK5 inhibitors in clinical settings.

This summary was initially drafted using artificial intelligence, then revised and fact-checked by the author.

## Introduction

Bone regeneration processes involve multiple growth factors and cells. Anabolic factors such as bone morphogenetic proteins (BMPs) and insulin-like growth factors enhance the osteoblast differentiation of mesenchymal stem cells (MSCs) and thus promote bone formation and regeneration^1^. In particular, recombinant human BMP2 (rhBMP2; BMP2) has been approved by the Food and Drug Association and is used as a therapeutic agent in clinical settings, where it can replace autografts^2^. However, to induce appropriate regeneration of bone, a supraphysiological dose of rhBMP2 is needed, which can lead to adverse effects such as inflammation, pain, adipose tissue formation, bone resorption, wound complications, and tumor formation^3–6^. Therefore, with the precise control of BMP2 signaling, appropriate bone regeneration with reduced adverse effects may be achieved.

BMP2 mediates biological functions by binding to type I and II serine/threonine kinase receptors, leading to the activation of intracellular signaling pathways, which are primarily categorized into the SMAD-dependent pathway, which includes SMAD1/5/9 (same as SMAD 1/5/8), and the SMAD-independent pathway, which involves p38, extracellular signal-regulated kinase (ERK), and Jun N-terminal kinase (JNK)^7^. The SMAD1/5/9 signaling pathway activated by BMP2 mediates osteoblast differentiation, as well as various biological functions, such as the formation of excessive adipose tissue, survival of retinal ganglion cells, and development of the nervous system^8–10^. On the other hand, the p38, ERK, and JNK pathways activated by BMP2 phosphorylate runt-related transcription factor 2 (RUNX2), the master transcription factor for osteoblast differentiation^7^. Notably, these pathways can also be activated by inflammatory cytokines such as tumor necrosis factor alpha (TNF-α) and interleukin-1-beta (IL-1β). Interestingly, these cytokines induce opposing effects on osteoblast differentiation^7^, indicating the possibility of varying BMP2 responses depending on microenvironmental conditions.

BMP2 also modulates Rac1, a Rho GTPase, and the activation of Rac1 attenuates osteoblast differentiation in C2C12 cells^11^. A dominant-negative mutant or chemical inhibitor of Rho-associated protein kinase (ROCK), a target of Rac1, can induce BMP2-induced osteoblast differentiation^12^. The existing evidence suggests that Rac1 may play a negative role in BMP2-induced osteoblast differentiation. However, the role of Rac1 in osteoblast differentiation or bone formation remains to be elucidated. In a previous study, genetic deletion of Rac1 in preosteoblasts via osterix-Cre attenuated osteoblast differentiation in vitro, with a reduction in bone mineral density as well as histomorphometric measures of osteoblast function^13^. In line with these results, the inhibition of Rac1 in certain osteoblastic cell lines, such as MC3T3-E1, does not enhance BMP2-mediated osteoblast differentiation^11^. These findings suggest that Rac1 regulation could be complicated, and an in-depth examination of upstream guanine nucleotide exchange factors (GEFs) could provide more detailed information for understanding the role of Rac1 in response to BMP2.

Several GEFs are required for activating Rac1 by catalyzing the exchange of bound GDP for GTP. A previous study reported that knockdown of T-lymphoma invasion and metastasis 1 (Tiam1), a Rac-specific GEF and a member of the Dbl family, could increase BMP2-induced alkaline phosphatase (ALP) activity^11^, indicating a negative role of Tiam in BMP2-induced osteoblast differentiation. Rac1 is also regulated by cell‒cell or cell‒extracellular matrix (ECM) contacts; thus, the differentiation of osteoblasts induced by BMP2 varies depending on cell density^14^. Suppression of CrkII, a regulator of Rac1, also promotes osteoblast differentiation^15^. These results suggest that even potent factors such as BMP2 may have different effects, depending on specific cellular conditions during the process of osteoblast differentiation.

In addition to the Dbl family of GEFs, DOCK family proteins also act as GEFs for Rac and/or Cdc42 but not for RhoA^16–18^. DOCK family GEFs contain the conserved Dock-homology region (DHR), and DOCK5 can physically interact with Rac1 through a special DHR2 (or CZH2) domain^19,20^. The DHR1 domain positions DOCK5 on phosphatidylinositol-3,4,5-phosphate-enriched membranes^21^. Furthermore, DOCK5-Rac1 is required for actin-ring formation in osteoclasts; as demonstrated in a previous study, mice lacking DOCK5-Rac1 presented reduced bone resorption by osteoclasts^21^. Considering the role of Rac1 in osteoblast differentiation, investigating the specific role of the DOCK5 GEF in the regulation of osteoblast differentiation and bone formation is essential. In this study, the role of DOCK5 in Rac1 activation, osteoblast differentiation, and bone regeneration and the underlying molecular mechanism were investigated using chemical inhibitors and a knockout (KO) mouse model.

## Materials and methods

### Osteoblast differentiation analysis

Mouse MC3T3-E1 cells were cultured in α-MEM without ascorbic acid supplemented with 10% FBS (Welgene), 100 U/mL penicillin, and 100 μg/mL streptomycin (P/S; Gibco BRL). Both mouse bone marrow mesenchymal stem cells (mBMSCs) and human bone marrow mesenchymal stem cells (hBMSCs) (PT-2501; Lonza, Basel, Switzerland) were cultured in α-MEM containing 10% FBS (Gibco BRL) and P/S. For osteogenic induction of MC3T3-E1 cells and mBMSCs, 10 μM beta-glycerophosphate and 50 μg/mL ascorbic acid were added to the medium (osteogenic induction (OS) medium). In the case of hBMSCs, 10 nM dexamethasone was added to the mouse OS medium. All the cells were incubated at 37 °C in a humidified atmosphere containing 5% CO_2_.

To induce osteoblast differentiation, MC3T3-E1 cells were seeded at a density of 5 × 10^4^/cm^2^ in 24-well plates. After 1 day, the cells were treated with OS medium for 3 days, followed by treatment with OS medium containing either 25 or 50 μM C21 (Sigma‒Aldrich) with 20 ng/mL rhBMP2 (BMP2 in the figures indicates rhBMP2; Cowell Medi). At 12 and 15 days following the initial OS medium treatment, the mineral deposits were stained with Alizarin Red S. For Alizarin Red S staining, the cells were washed with 1× PBS and fixed with 70% ethyl alcohol for 20 min at room temperature. Following fixation, the cells were rinsed twice with distilled water, and Alizarin Red S (40 mM, pH 4.2) was applied for 10 min. Nonspecific staining was removed with water five times.

For the osteoblast differentiation of hBMSCs, the cells were seeded at a density of 1 × 10^4^ cells/cm^2^ in 24-well plates. The next day, OS medium was added for 3 days. Starting from the 3rd day of OS treatment, 50 ng/mL rhBMP2 combined with either 10 or 25 μM C21 was added to the medium for further differentiation. The OS medium containing rhBMP2 and C21 was replaced every 3 days during the OB differentiation process.

BMSCs isolated from mouse bone marrow as previously described^22^ were seeded (2.5 × 10^4^ cells) in 24-well plates. Upon reaching 50% confluence, OS medium or OS medium supplemented with 50 ng/mL rhBMP2 was added. On Day 6, the cells were stained with Alizarin Red S solution.

### Western blotting and phosphorylation

To examine the phosphorylation levels of signaling molecules in MC3T3-E1 cells, 4.8 × 10^5^ cells were seeded in 35 mm dishes. After 24 h, the medium was replaced with one containing 0.3% FBS and incubated for another 24 h. For OS, either vehicle or 100 μM C21 was added (for the 0 min sample, no addition), and after 5, 15, 30, and 60 min, the stimulation was terminated with the addition of ice-cold PBS twice. For OS + BMP2, MC3T3-E1 cells were seeded and cultured in OS medium for 3 days, followed by 24 h of treatment in OS medium supplemented with 0.3% FBS. The cells were subsequently treated with 50 ng/mL rhBMP2 and either vehicle or 100 μM C21 at each time point.

In addition, hBMSCs (1 × 10^5^ cells) and mBMSCs (2 × 10^5^ cells) were seeded in 35 mm dishes and cultured in OS medium for 3 days. C21 and BMP2 were added at the same time points as those used for MC3T3-E1 cells.

For cell lysis, after washing with ice-cold 1× PBS, 1× gel loading buffer (GLB; 50 mM Tris-HCl pH 6.8, 2% SDS, 6% glycerol, and 0.01% bromophenol blue) was used. The cell lysates were then sonicated for 43 s with a 1 s on/2 s off cycle and 20% amplitude using a microprobe sonicator (Qsonica). The protein concentration in the lysates was measured via a BCA protein assay kit (Thermo Fisher Scientific), and 5% β-mercaptoethanol was added to 30 μg of each protein sample. Western blotting was performed as described previously^23^. Specific antibodies against phospho-ERK1/2 (9101S), EKR1/2 (9102S), phospho-JNK (9251S), JNK (9252S), phospho-p38 (9211S), p38 (9212S), phospho-AKT (4058S), AKT (9272S), phospho-TAK1 (9339S), MKK3 (5674S), phospho-MKK3/6 (9231S), SMAD1 (9743S), phospho-SMAD1/5/9 (13820S), phospho-MEK1/2 (9154S), MEK1/2 (9122S), and BSP (5468S) were purchased from Cell Signaling Technology, Inc. Antibodies against RUNX2 (sc-101145), OSX (SP7; ab55252), TAK1 (A12022), and β-actin (A5441) were purchased from Santa Cruz Biotechnology, Abcam, ABclonal, and Sigma‒Aldrich, respectively.

### ALP staining and activity assay

MC3T3-E1 cells were seeded in 24-well plates and cultured for osteoblast differentiation as described above. To observe the effect of Rac1 inhibition, the cells were pretreated with 50 μM NSC23766 (MedChemExpress) for 14 h, followed by treatment with DMSO or 50 μM C21 and 20 ng/mL rhBMP2 in OS medium. On the 7^th^ day of differentiation, the cells were stained with an ALP staining kit (EMD Millipore Corp.) following the manufacturer’s instructions. To determine the effect of C21 on TAK1 signaling, the cells were pretreated with 10 μM HS-276 (TAK1 inhibitor; Sigma‒Aldrich) in OS medium for 24 h and then were cotreated with 2 μM HS-276 and 50 ng/mL rhBMP2 with/without 25 μM C21 in OS medium. To measure ALP activity, on Day 7, the cells were washed with ice-cold 1× PBS twice, and the activity was measured according to the manufacturer’s instructions (Abcam). The measured pNPP concentration was normalized to the protein concentration of each sample.

### Rac1 activity assay

MC3T3-E1 cells were seeded in 12-well plates with culture medium, which was subsequently replaced with OS medium for 3 days. The medium was subsequently changed to OS medium containing 0.3% FBS for starvation (24 h). The cells were treated for 1 h as follows: OS + vehicle, OS + rhBMP2 (20 μg/mL), and OS + rhBMP2 + C21 (either 25 μM or 50 μM in 0.3% FBS). Then, cell lysates were prepared, and Rac1 activity was analyzed according to the manufacturer’s instructions (Cytoskeleton Inc.).

### Dock5 null mice

Dock5 deletion was achieved by gene trap insertion of the beta-Geo cassette between exons 1 and 2 (Supplementary Fig. 1), resulting in the complete deletion of DOCK5. The null mice were produced by the Texas Institute for Genomic Medicine (Houston).

### Micro-CT analysis

The specimens were fixed in 10% formalin/PBS (pH 7.4) for 24 h at 4 °C, followed by multiple washes with PBS. Micro-CT scans of the tibial, calvarial, and ectopic bones were subsequently performed via a SkyScan 1272 scanner (Bruker) as described previously^23^.

### Histomorphometry and calcein assay

Skeletons from 9-week-old mice were prepared as described previously^22^. The tibiae and lumbar vertebrae were fixed in 10% formalin at 4 °C for 24 h, decalcified in 0.5 M EDTA (pH 8.0) at 4 °C for 2–3 weeks, and embedded in paraffin. The paraffinized tibial samples were cut in the sagittal plane to a thickness of 6 μm. These sections were stained with H&E and Masson’s trichrome stain (Polyscience) following the manufacturer’s instructions. The calvarial bone and ectopic bone were also processed following the same procedures. The stained areas were examined via a bright-field light microscope (Leica Microsystems GmbH). In addition, von Kossa staining was performed on the fixed lumbar vertebrae as described previously^22^. To analyze the MAR, 8-week-old mice were injected with calcein (20 mg/kg) on Days 1 and 6. Two days after the second injection, the mice were sacrificed, processed, and analyzed as described above.

### Mouse care and animal experiments

Animal experiments were conducted in accordance with the approved guidelines of Kyungpook National University (No. 2022-0459, 2023-0381, and 2024-0007). *Dock5* KO mice were housed under specific pathogen-free conditions at 22–24 °C and 50–60% humidity with a 12 h light/dark cycle and free access to water and food pellets. For euthanasia, a CO_2_ flow rate of 40–60% of the chamber volume per minute was used. BMMs were obtained from wild-type BL6 male mice aged 4–6 weeks, which were housed under similar conditions. Euthanasia was carried out via the intraperitoneal injection of 480 mg/kg avertin, followed by cervical dislocation under anesthesia.

A mouse calvarial defect model was created using WT (n = 6) and *Dock5* KO (n = 10) mice aged 6–7 weeks as described previously^24^. The mice were anesthetized via an intraperitoneal injection of 240 mg/kg avertin. A midline incision and periosteum retraction in the parietal region were made to expose the calvarial bone. Using a 3 mm trephine burr on a low-speed dental handpiece (Surgic XT) with sterile PBS irrigation, a circular defect was created in the left parietal bone. The defect was recovered with the periosteum, and the skin was sutured with 5–0 soluble silk sutures. No adverse events were observed during the experiments, and the mice were sacrificed after 8 weeks.

For the mouse calvarial ectopic bone formation model, the procedure for exposing the bony area was the same as that described above. A 2 mm diameter collagen sponge soaked in 1 µg of rhBMP2 was placed on the calvarial bone. The periosteum was then repositioned, and the skin was sutured. The mice were euthanized after 4 weeks. For subcutaneous implantation, a 3 mm diameter collagen sponge was used for the groups treated with either 1.5 μg of rhBMP2 with DMSO or 1.5 μg of rhBMP2 with 100 μg of C21. Both agents were mixed in a tube, and 3 μL of each mixture was used for soaking the collagen sponge, which was then implanted in the subcutaneous area of 6-week-old C57BL/6 J male mice. At 6 weeks after implantation, the inserted scaffolds were isolated and analyzed. The euthanasia procedure was performed with 480 mg/kg avertin, as described above.

### Immunohistochemistry

Cryosectioning, blocking, and antibody dilution were performed as described in the Supplementary Information.

### Statistical analysis

All the experiments were repeated at least three times. The experimental results are presented as the mean ± standard error of the mean (SEM). The data were analyzed via two-tailed unpaired Student’s t test or one-way analysis of variance (ANOVA) with Tukey’s multiple comparison post hoc test. A *p* value of less than 0.05 was considered statistically significant.

## Results

### Inhibition of DOCK5 with C21 enhances BMP2-mediated osteoblast differentiation

**Fig. 1:**
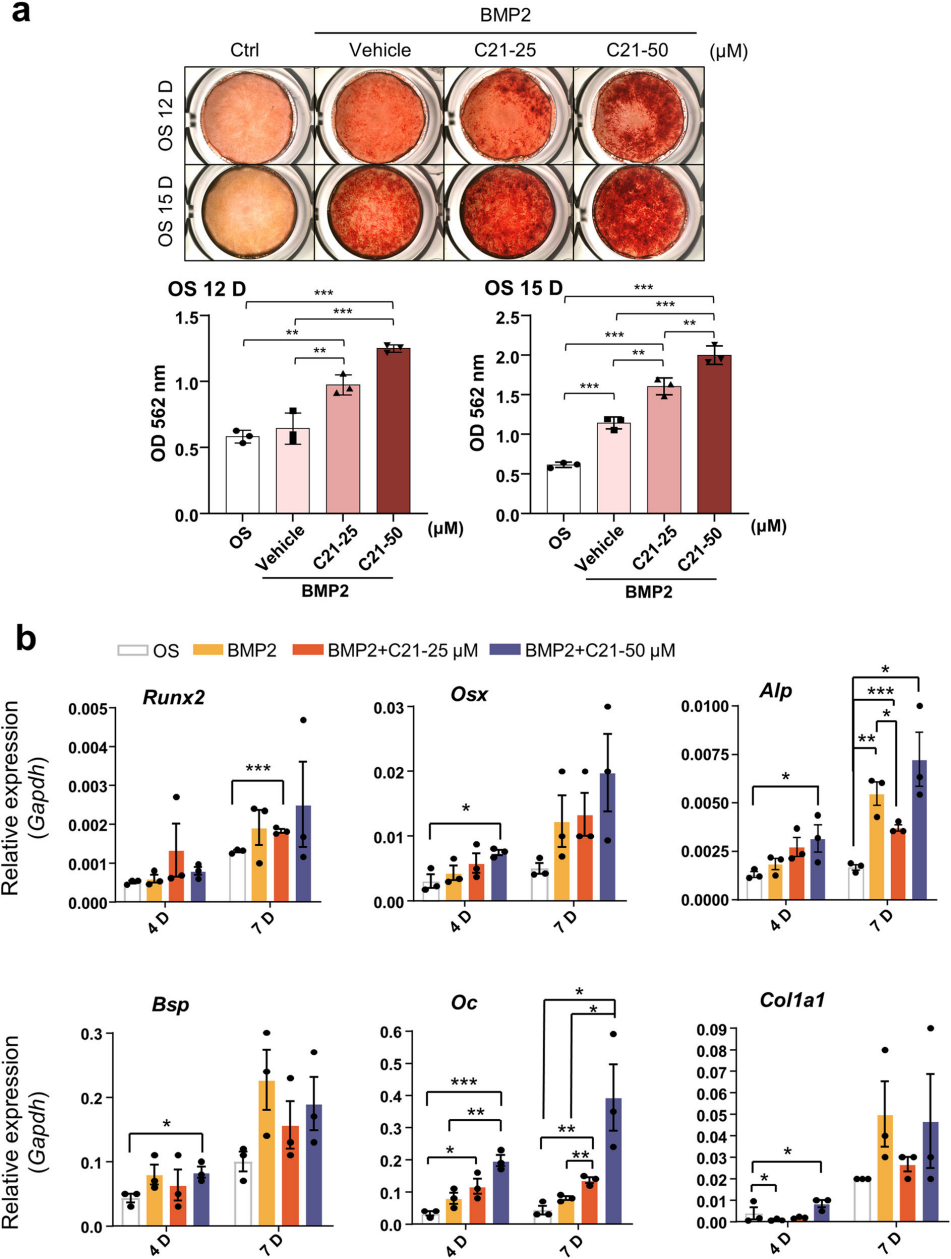

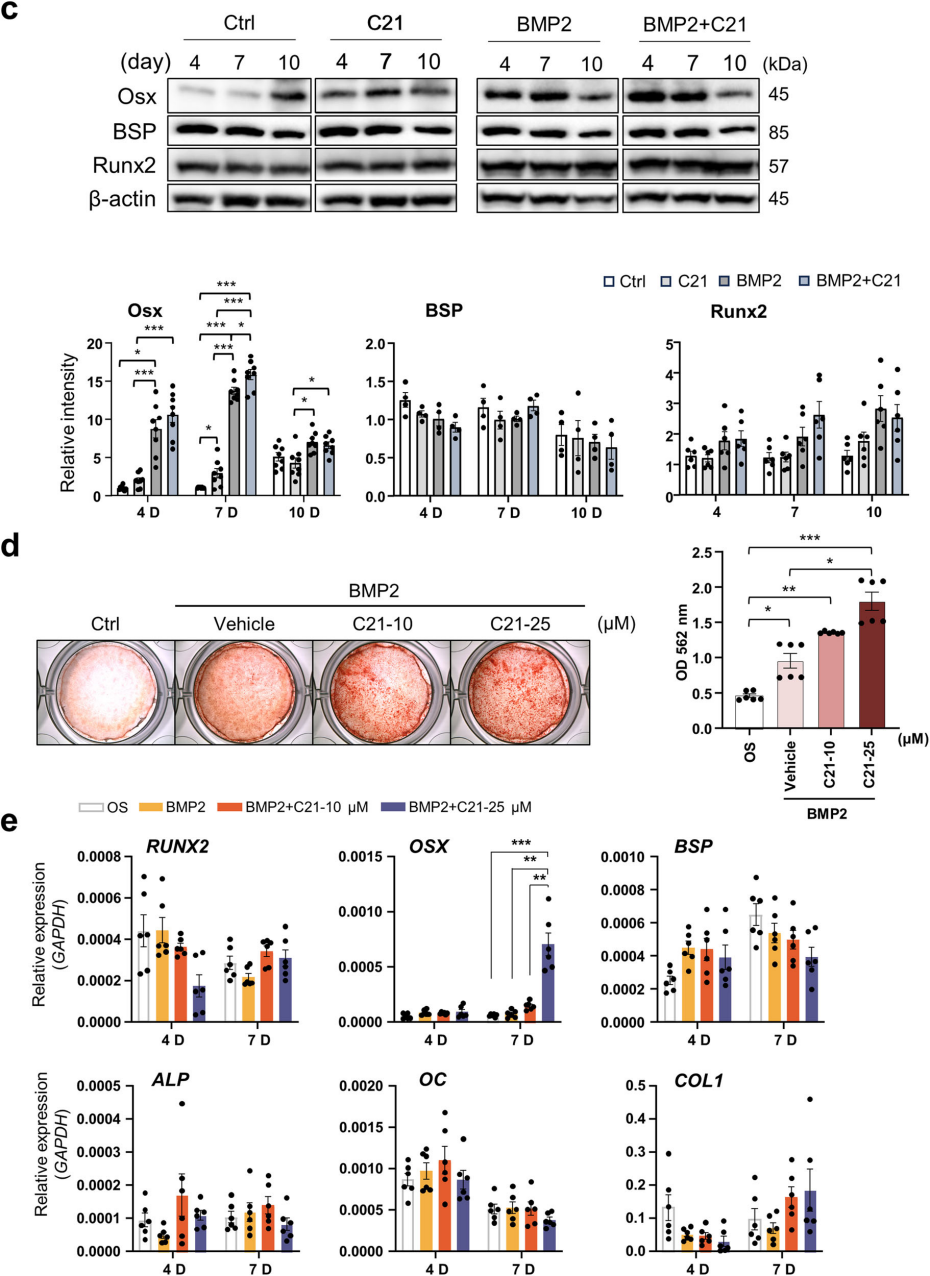
Effects of C21 on osteoblast differentiation. **a** MC3T3-E1 cells were treated with rhBMP2 and 25 or 50 μM C21 for 12 or 15 days. Mineral deposition was visualized with Alizarin Red S solution (n = 3). **b** RT‒qPCR analysis of the expression of osteogenesis-specific marker genes in cells undergoing osteoblast differentiation on Days 4 and 7 (n = 3). The expression of each gene was normalized to *Gapdh* expression. **c** Western blotting for the protein expression of OSX, BSP, and RUNX2 on Days 4, 7, and 10 during osteoblast differentiation (n = 4 ~ 8). **d** hBMSCs treated with 50 ng/mL rhBMP2 and 10 or 25 μM C21 for 12 days. Mineral deposition was visualized with Alizarin Red S solution (n = 6). **e** Analysis of the expression of osteoblast-specific marker genes in hBMSCs undergoing osteoblast differentiation on Days 4 and 7 (n = 6). *p < 0.05, **p < 0.01, and ***p < 0.001 as analyzed by one-way ANOVA with Tukey’s multiple comparison post hoc test. rhBMP2 recombinant human bone morphogenetic protein 2, RT‒qPCR reverse transcription‒quantitative polymerase chain reaction, *Gapdh* glyceraldehyde-3-phosphate dehydrogenase, OSX/*Osx* osterix, BSP/*Bsp* bone sialoprotein, RUNX2/*Runx2* runt-related transcription Factor 2, *Alp* alkaline phosphatase, *Oc* osteocalcin, *Col1a1* collagen, type I, alpha 1, *and* hBMSCs human bone marrow mesenchymal stem cells.

To examine the role of DOCK5 in osteoblast differentiation, MC3T3-E1 cells were treated with C21 at a concentration of 25 or 50 μM, and osteoblast differentiation was analyzed. C21 was not cytotoxic in the range of 1 to 1000 μM in MC3T3-E1 cells and 5 to 100 μM in hBMSCs (Supplementary Fig. 2). When the cells were treated with C21 in the presence of OS medium, an increase in mineral formation was observed at a concentration of 100 μM (data not shown). DOCK5 is known to be involved in cell migration and actin polymerization in osteoclastic cells of the myeloid lineage^25^. To allow MC3T3-E1 cells to grow and migrate adequately during the initial stage of commitment to osteoblasts, the cells were incubated with OS medium for 2–3 days, and rhBMP2 (20 ng/mL) with or without C21 was added for another 12 and 15 days. As shown in Fig. 1, mineralization significantly increased in a concentration-dependent manner from the 12th and 15th days of differentiation (Fig. 1a). To confirm that C21 induced osteoblast differentiation, the expression of osteogenic marker genes was examined, and the expression of most marker genes appeared to be increased. In particular, the expression of osterix (*Osx)* and bone sialoprotein (*Bsp)* was greater on Day 4 in cells treated with BMP2 and 50 μM C21 than in control cells. The expression of osteocalcin (*Oc)* and collagen, type I, alpha 1 (*Col1a1)* was also markedly increased in cells treated with BMP2 and 50 μM C21 compared with that in cells treated with BMP2 alone (Fig. 1b). The expression of specific protein markers for osteoblast differentiation was examined. OSX protein expression was significantly greater after treatment with C21 and BMP2 than after treatment with BMP2 alone. Interestingly, the expression of OSX was induced even without treatment with BMP2, implying that the inhibition of DOCK5 could increase the expression of osteoblast marker genes/proteins (Fig. 1c). In hBMSCs, the effect of C21 on osteoblast differentiation was also examined. Cotreatment with C21 (0, 10, or 25 μM) and BMP2 (50 ng/mL) synergistically and dose-dependently increased mineral deposition, as assessed by Alizarin Red S staining (Fig. 1d). Osteoblast differentiation was also confirmed on the basis of the expression of osteoblast marker genes. Notably, the *Osx* gene was significantly induced (Fig. 1e). Mineral deposition and osteoblast differentiation in mBMSCs were also induced by BMP2 and C21 (Supplementary Fig. 3). These results suggest that the inhibition of DOCK5 by C21 could synergistically induce osteoblast differentiation with BMP2.

### Genetic deletion of *Dock5* enhances bone formation via increased osteoblast differentiation

**Fig. 2:**
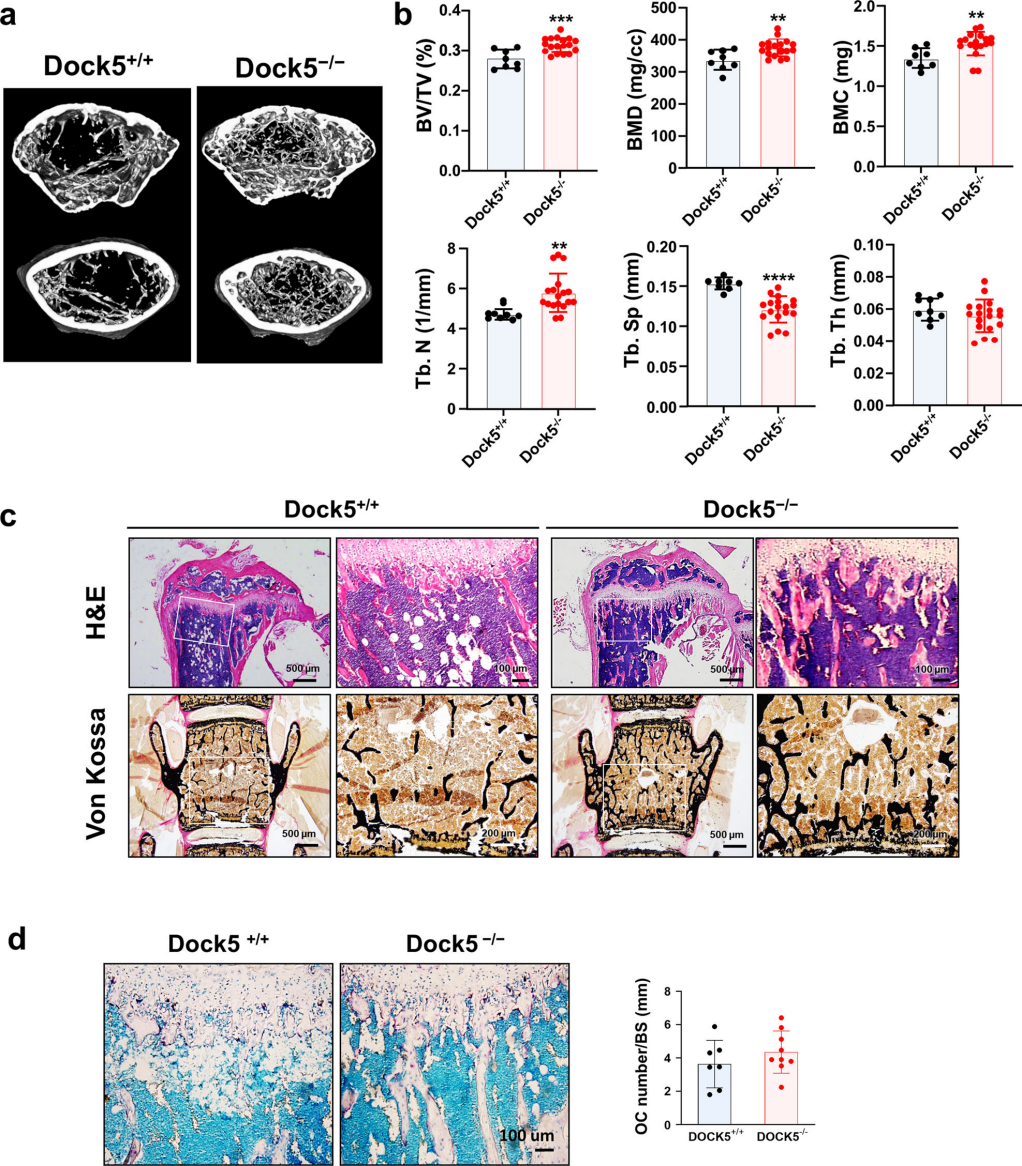

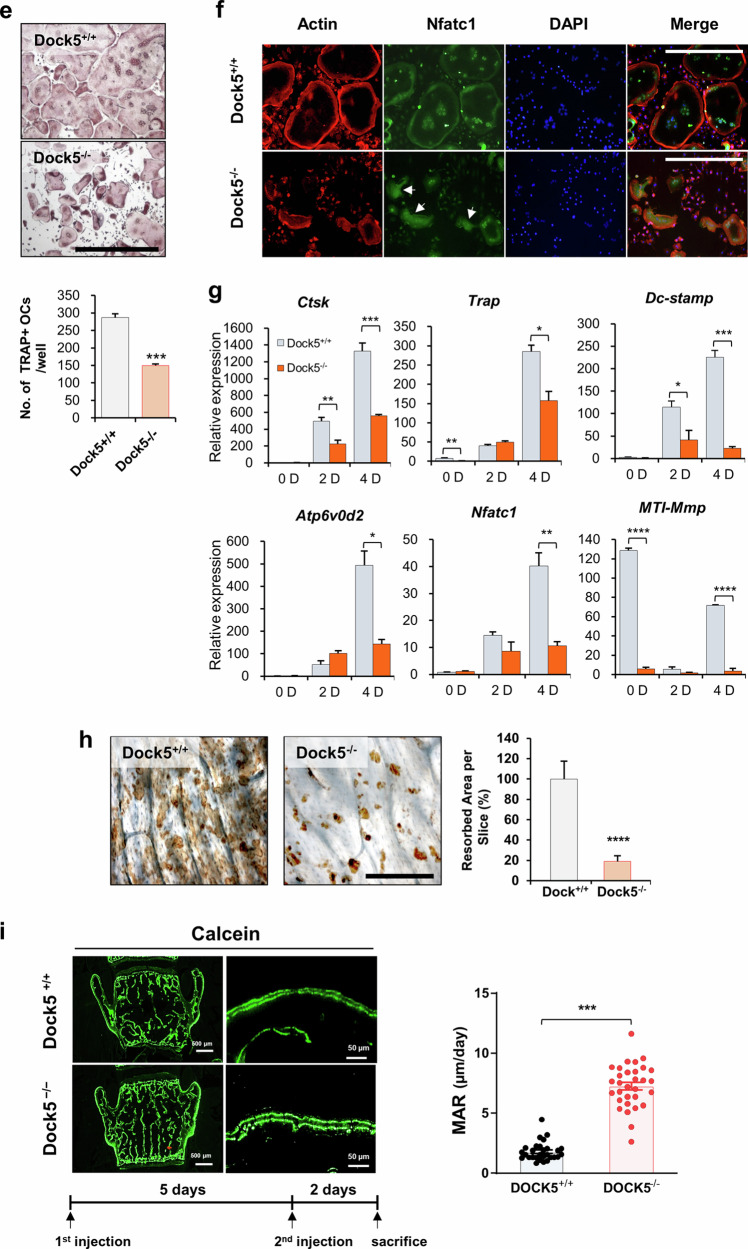
Characteristics of the bones of *Dock5* KO mice. **a** Representative micro-CT images showing the trabecular area in the transverse plane of WT and *Dock5* KO mice. **b** Quantitative analysis of the bone parameters of WT and *Dock5* KO mice. The bone volume per tissue volume (BV/TV), bone mineral density (BMD), bone mineral content (BMC), trabecular number (Tb.N), trabecular separation (Tb.Sp), and trabecular thickness (Tb.Th) were analyzed (n = 8 for WT and 18 for KO). **c** Hematoxylin and eosin (H&E) staining of the tibial trabecular bone and silver staining of the lumbar spine (von Kossa). The boxed areas are shown at a higher magnification next to the low-magnification images. **d** TRAP staining of the tibial trabecular bone and the number of TRAP-positive osteoclasts. **e** Osteoclast differentiation of BMMs from *Dock5* KO and WT mice under M-CSF and RANKL treatment following TRAP staining and the number of TRAP-positive osteoclasts. **f** Staining of cells with rhodamine-phalloidin (red), DAPI (blue), and an anti-Nfatc1 antibody (green) to detect actin, nuclei, and Nfatc1, respectively. **g** Quantification of osteoclast-specific gene expression via RT‒qPCR. **h** Resorption pit formation assay. BMMs were seeded on bone slices and cultured under osteoclast induction conditions for 5 days, followed by staining with peroxidase-conjugated wheat germ agglutinin to observe resorption pits (n = 3). The resorption pit area per bone slice was measured via ImageJ software. The statistical significance of the expression of each gene was analyzed on the same day only. *p < 0.05, **p < 0.01, ***p < 0.001, and ****p < 0.0001 as analyzed by two-tailed unpaired Student’s t test. **i** Calcein labeling of 8-week-old mice on Days 1 and 6. Two days after the second injection, the mice were sacrificed and analyzed. Representative calcein-labeled images of the lumbar spine from WT (upper panel) and *Dock5* KO (lower panel) mice are shown on the left. Magnified images are shown on the right. The mineral apposition rate (MAR) was calculated (n = 29 for WT and 31 for KO). *p < 0.05, **p < 0.01, and ***p < 0.001 as analyzed by one-way ANOVA with Tukey’s multiple comparison post hoc test or unpaired Student’s t test. *Dock5* dedicator of cytokinesis 5, KO knockout, micro-CT microcomputed tomography, WT wild-type.

To investigate the in vivo or genetic role of *Dock5* in osteoblast differentiation and bone formation, *Dock5* KO mice were generated by gene trapping immediately following exon 1 of the *Dock5* gene (Supplementary Fig. 1a), resulting in translational termination at the SH3 domain of the DOCK5 protein (Supplementary Fig. 1b), and bone formation was analyzed. We first characterized the skeletal features of *Dock5* KO mice. Microcomputed tomography (micro-CT) analysis of long bones (7–9 weeks old) revealed a 13% increase in the bone volume per tissue volume (BV/TV), an 11% increase in the bone mineral density (BMD), a 17% increase in the bone mineral content (BMC), a 28% increase in the trabecular number (Tb.N), and a 36% decrease in the trabecular separation (Tb.Sp) in *Dock5* KO mice compared with those in WT mice (Fig. 2a, b). Hematoxylin and eosin (H&E) and von Kossa staining of the tibia and spine revealed an increase in bone mass (Fig. 2c). This increase in bone mass could be attributed to reduced osteoclast activity and heightened osteoblast activity. To further investigate this outcome, we evaluated osteoclast differentiation and the osteoblastic bone mineral apposition rate (MAR). TRAP staining of tibial sections revealed no significant difference in the number of osteoclasts between WT and *Dock5* KO mice (Fig. 2d). However, we observed significant suppression of osteoclast differentiation in bone marrow macrophages (BMMs) from *Dock5* KO mice, accompanied by a marked decrease in bone-resorbing activity (Fig. 2e–h). Moreover, as shown in Fig. 2i, double calcein labeling revealed increased levels of newly calcified bone and elevated MAR in the spines of *Dock5* KO mice compared with those of WT mice (Fig. 2i). These findings strongly suggest that enhanced osteoblastic bone formation could also contribute to the increased bone mass observed in *Dock5* KO mice.

**Fig. 3:**
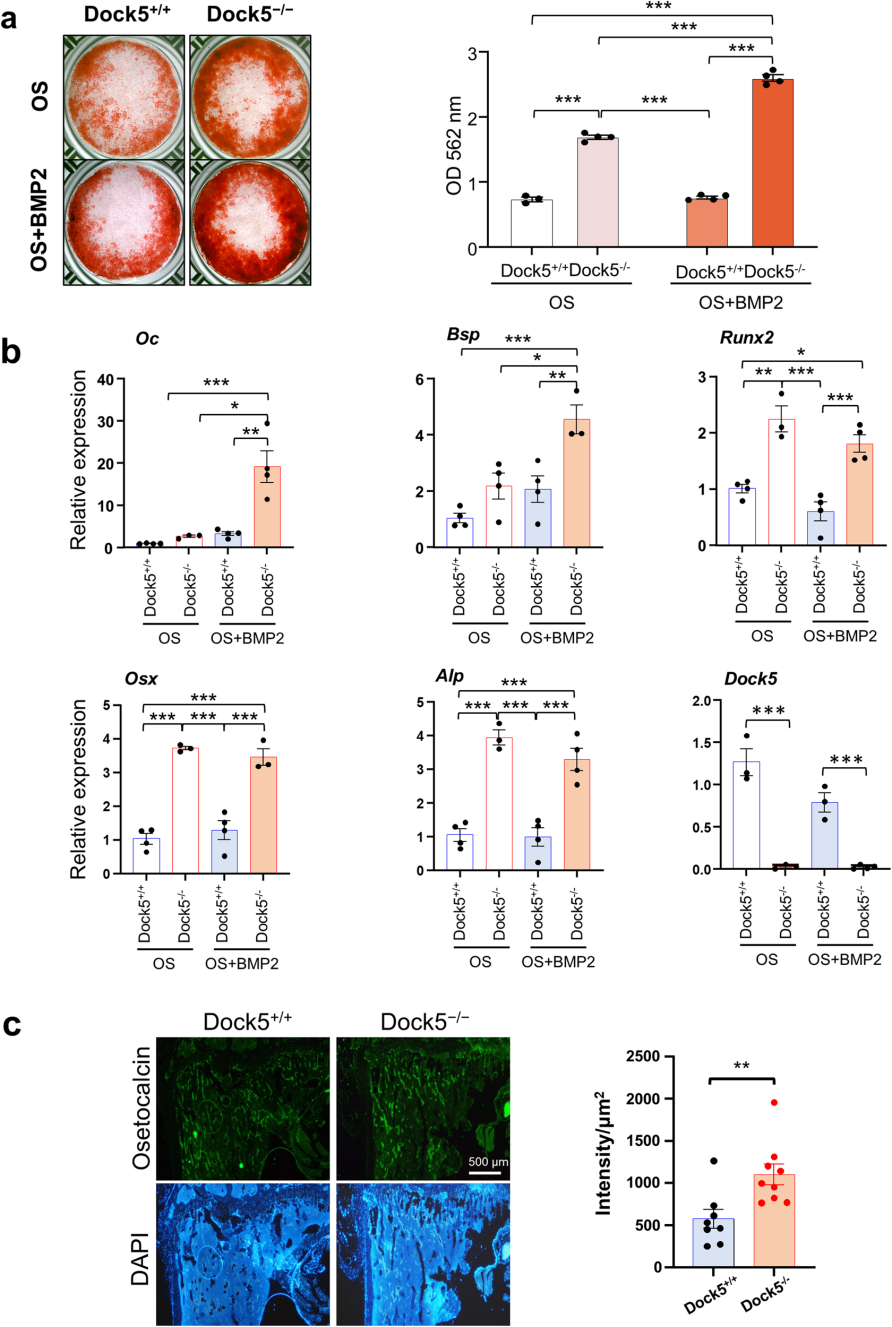
Effect of *Dock5* deletion on osteoblast differentiation. **a** Osteoblast differentiation of mBMSCs isolated from WT and *Dock5* KO mice induced with OS or OS + BMP2. Mineral deposition was assessed by Alizarin Red S staining (n = 4). **b** RT‒qPCR analysis of the expression of osteoblast-specific marker genes on Day 3 (n = 4). **c** Immunohistochemistry for osteocalcin in the tibial trabecular bone (n = 8). *p < 0.05, **p < 0.01, and ***p < 0.001 as analyzed by one-way ANOVA with Tukey’s multiple comparison post hoc test or unpaired Student’s t test. *Dock5*: dedicator of cytokinesis 5, KO: knockout, WT: wild-type, mBMSCs: mouse bone marrow mesenchymal stem cells, OS osteogenic induction, BMP2 bone morphogenetic protein 2, *Oc* osteocalcin, *Bsp* bone sialoprotein, *Runx2* runt-related transcription Factor 2, *Osx* osterix, *Alp* alkaline phosphatase, RT‒qPCR reverse transcription‒quantitative polymerase chain reaction.

To further investigate the effect of *Dock5* absence on osteoblast differentiation independent of osteoclast activity, BMSCs were isolated from the bone marrow of *Dock5* KO and WT mice, and osteoblast differentiation was induced as follows: OS or OS + BMP2. In response to BMP2, mineral deposition by BMSCs derived from *Dock5* KO mice was markedly increased by Day 6 (Fig. 3a). Furthermore, the expression levels of specific markers associated with osteoblast differentiation, such as *Oc*, *Bsp*, *Runx2*, *Osx*, and *Alp*, were significantly elevated in the BMSCs from *Dock5* KO mice compared with those from WT mice (Fig. 3b). Immunostaining for OC protein and intensity analysis (per μm^2^) also revealed a significant increase in trabecular bone in the tibia of *Dock5* KO mice (Fig. 3c). These results suggest that the complete deletion of *Dock5* could enhance osteoblast differentiation synergistically with BMP2, even in the absence of osteoclast activity.

### *Dock5* genetic deletion and chemical inhibition of DOCK5 enhance bone regeneration in calvarial defect and ectopic bone formation mouse models

**Fig. 4:**
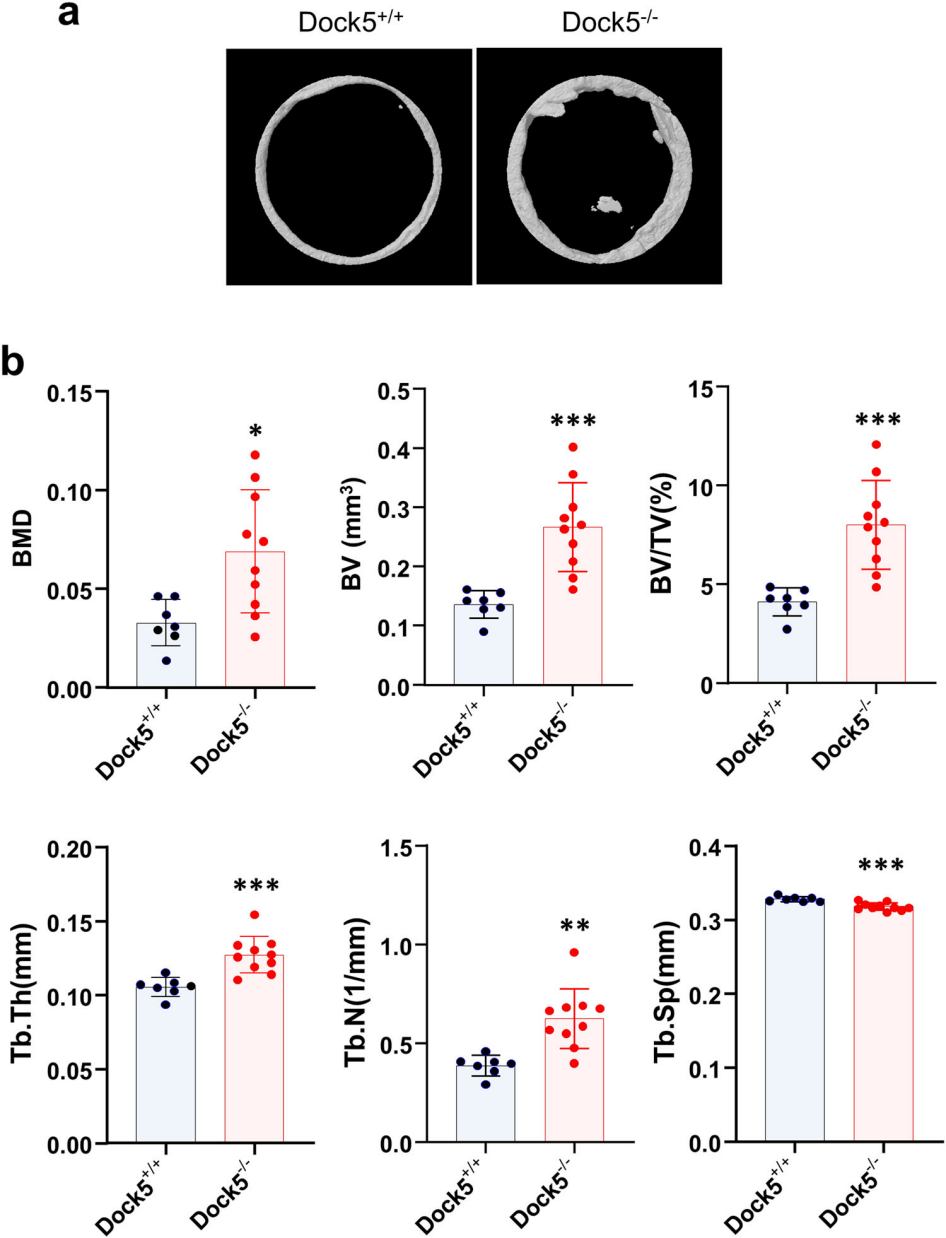

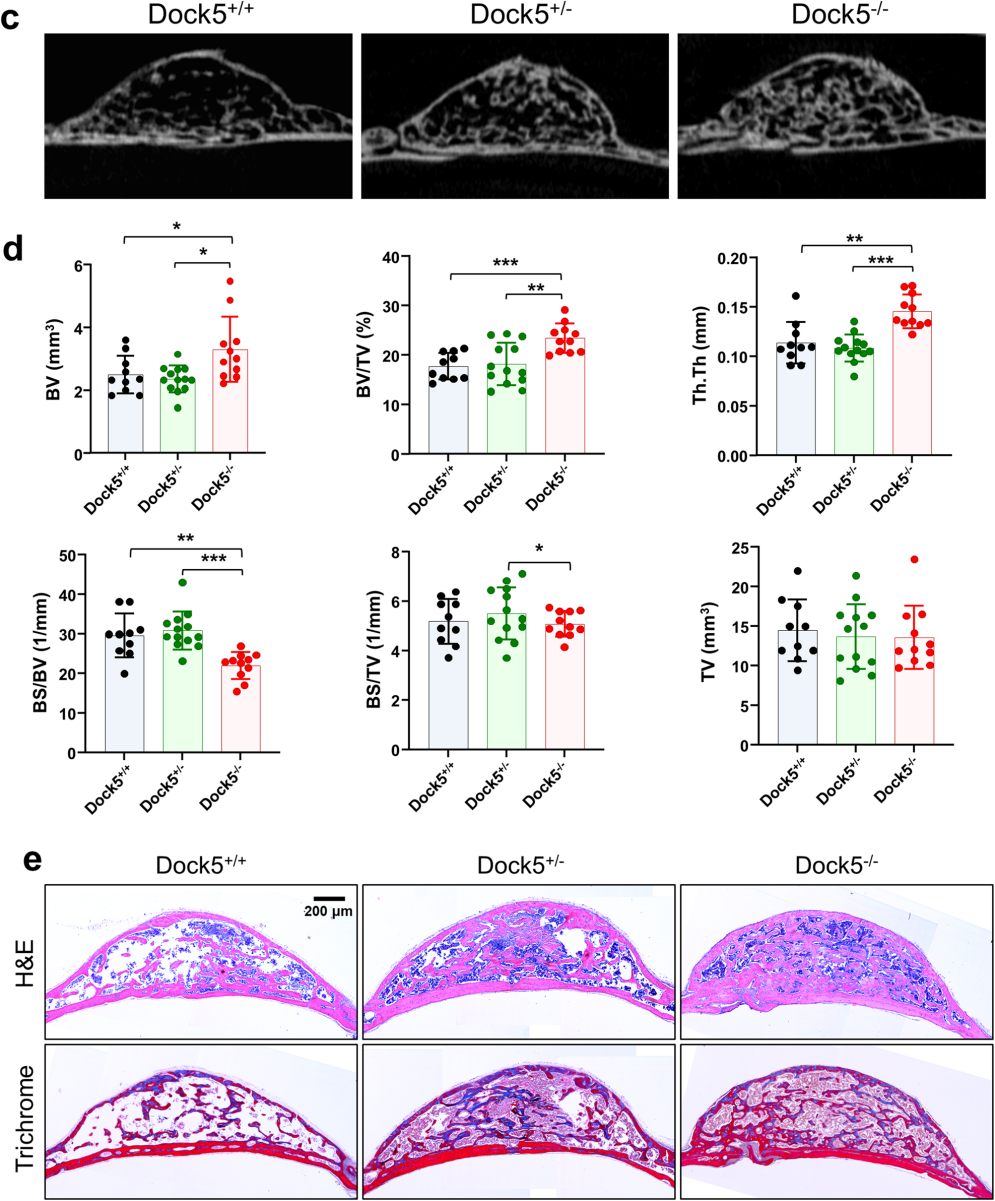

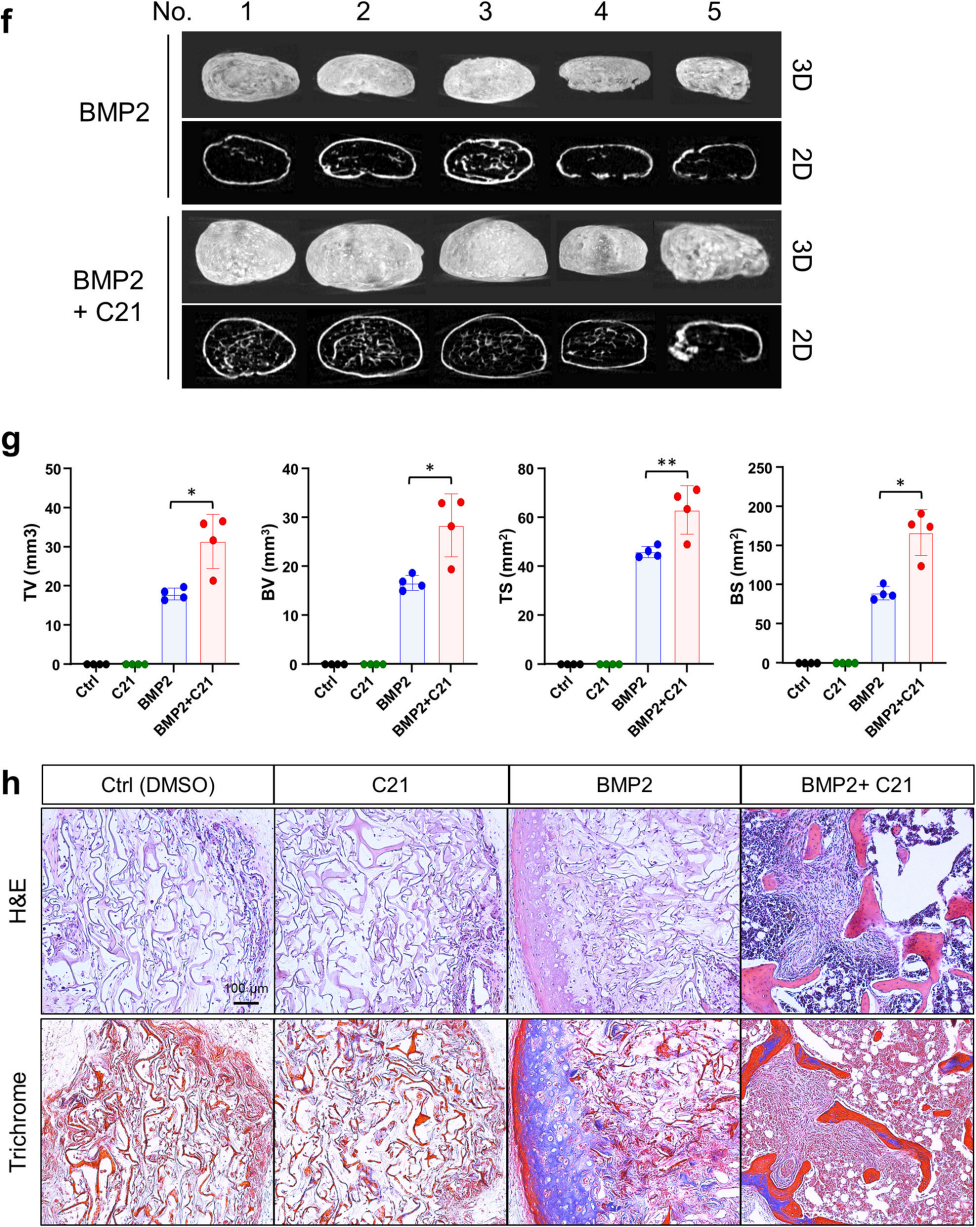
Effects of *Dock5* genetic deletion and chemical inhibition of DOCK5 on bone regeneration in animal models. **a** Micro-CT image of the regeneration of calvarial defects in WT and *Dock5* KO mice. Representative images are shown. **b** Analysis of bone parameters via micro-CT (n = 7~10 per group). **c** Ectopic bone formation under the calvarial periosteum in WT, *Dock5* heterozygous, and *Dock5* KO mice induced with BMP2. Representative micro-CT images are shown. **d** Analysis of bone parameters according to genotype group (n = 10~13). *p < 0.05, **p < 0.01, and ***p < 0.001 as analyzed by one-way ANOVA with Tukey’s multiple comparison post hoc test. **e** H&E and Masson’s trichrome staining of calvarial ectopic bones in sections. Scale bar = 200 μm. **f** Subcutaneous ectopic bone formation induced by BMP2 or BMP2 + C21 in C57BL/6 mice. Micro-CT images of ectopic bones are shown (n = 5). **g** Analysis of bone parameters from micro-CT according to the treatment group. *p < 0.05 and **p < 0.01 as analyzed by one-way ANOVA with Tukey’s multiple comparison post hoc test. **h** H&E (upper) and Masson’s trichrome (lower) staining of ectopic bones. Scale bar = 100 μm. *Dock5* dedicator of cytokinesis 5, KO knockout, WT wild-type, micro-CT microcomputed tomography, BMP2 bone morphogenetic protein 2, H&E hematoxylin and eosin, BMD bone mineral density, TV tissue volume, BV bone volume, BV/TV bone volume per tissue volume, Tb.Th trabecular thickness, Tb.N trabecular number, Tb.Sp trabecular separation, TS tissue surface, BS bone surface.

As the inhibition of DOCK5 or the deletion of *Dock5* promoted osteoblast differentiation in both hBMSCs and mBMSCs as well as in osteoblastic cells, we investigated the in vivo role of *Dock5* in bone regeneration. A typical mouse model used in bone regeneration is the calvarial defect model. Accordingly, a 3 mm defect was surgically created on the calvaria of each mouse, and bone regeneration was assessed after 8 weeks following the protocol outlined in the Materials and Methods section. Microcomputed tomography (micro-CT) analysis revealed a significant increase in bone regeneration at the marginal zone of calvarial defects in *Dock5* KO mice (Fig. 4a). The BMD, bone volume (BV), and BV/TV were approximately doubled in *Dock5* KO mice compared with WT mice. The trabecular thickness (Tb.Th) was increased by 1.2-fold, and the Tb.N was increased by 1.6-fold. Tb.Sp significantly decreased by 4% (Fig. 4b).

However, it is important to consider that in the highly invasive calvarial defect model, the large influx of blood cells, including myeloid lineage cells that can serve as precursors to osteoclasts, may contribute to bone regeneration. To minimize the influence of osteoclasts, a calvarial ectopic bone formation model was employed. In this model, a 2 mm collagen sponge soaked with BMP2 (1 μg) was placed under the calvarial periosteum to induce bone formation via periosteal MSCs. After 4 weeks, the mice were sacrificed, and the ectopic bones formed were examined. Overall, micro-CT analysis revealed that the size of the ectopic bones formed was similar across groups; however, in comparison with *Dock5* heterozygous (HE) or WT mice, *Dock5* KO mice presented an increase in trabecular bone within the ectopically formed bone (Fig. 4c). Compared with those of WT mice, the bone parameters of the ectopic bones of *Dock5* KO mice were 32% greater in BV, with a similar increase (32%) in BV/TV compared with those of WT mice and a 29% greater BV/TV than those of *Dock5* HE mice (Fig. 4d). Tb.Th was also significantly increased by 28% in *Dock5* KO mice compared with WT mice and by 29% in HE mice (Fig. 4d). Histological analysis via H&E staining revealed greater trabecular bone formation in *Dock5* KO mice than in *Dock5* HE or WT mice. The maturation of newly formed bone was confirmed by trichrome staining (Fig. 4e).

Furthermore, ectopic bone regeneration in a subcutaneous model was used to demonstrate the role of DOCK5 in osteoblast differentiation and bone formation. Subcutaneous implantation with a collagen sponge soaked with BMP2 or BMP2 + C21 was conducted as described in the Materials and Methods section. The results revealed that larger ectopic bones were produced with BMP2 + C21 than with BMP2 alone (Fig. 4f). The tissue volume (TV), BV, tissue surface (TS), and bone surface (BS) were also significantly increased by C21 (Fig. 4g). Histology revealed that ectopic bones formed with BMP2 + C21 had a greater number of trabeculae (Fig. 4h), indicating that DOCK5 inhibition by C21 strongly enhanced BMP2-induced ectopic bone formation. Taken together, the chemical inhibition of DOCK5 and genetic ablation of *Dock5* could enhance in vivo bone formation and regeneration by potentiating osteoblast differentiation.

### *Dock5* deletion or inhibition of DOCK5 enhances BMP2-induced phosphorylation of the MKK3/6 and p38 pathways

**Fig. 5:**
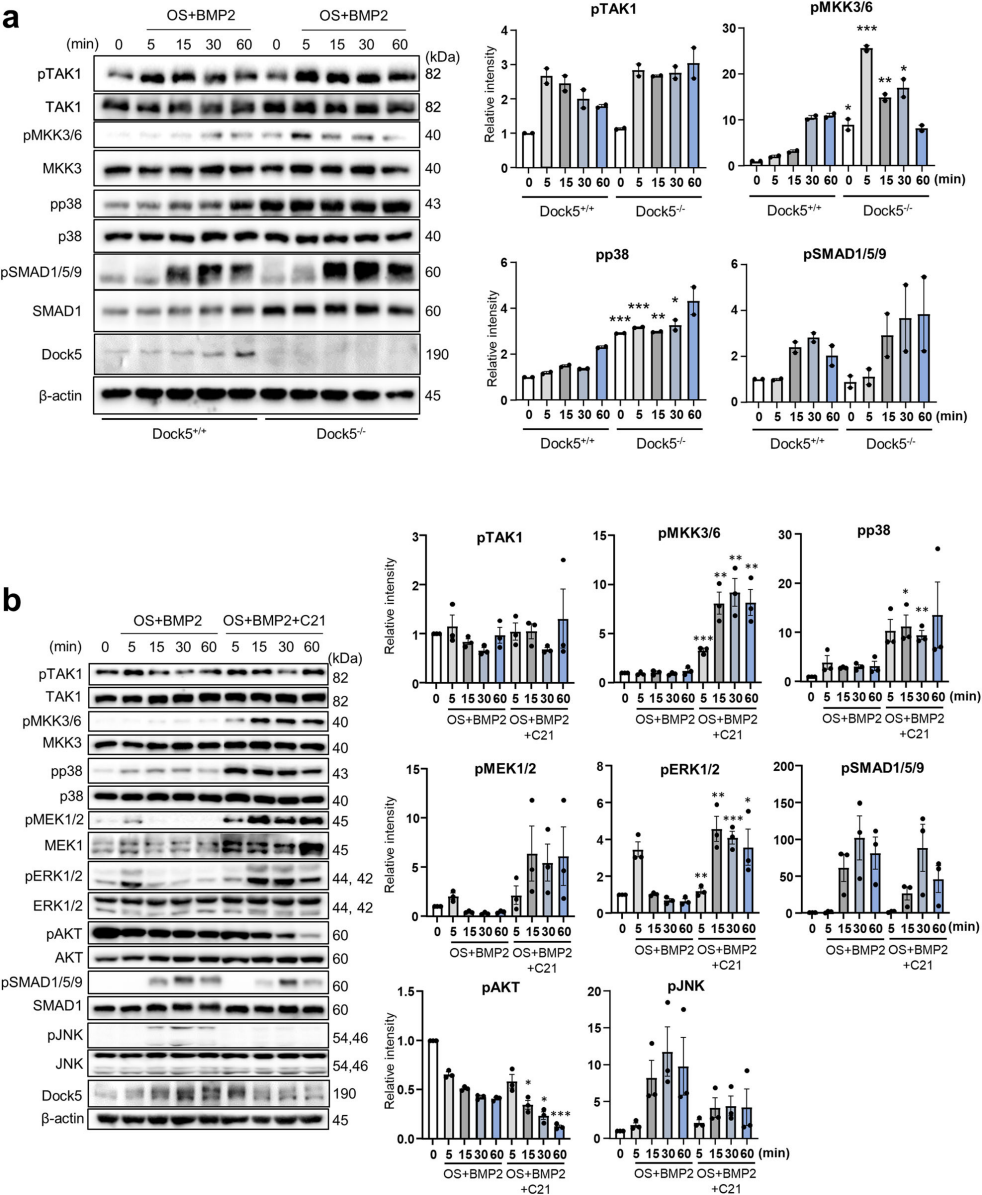

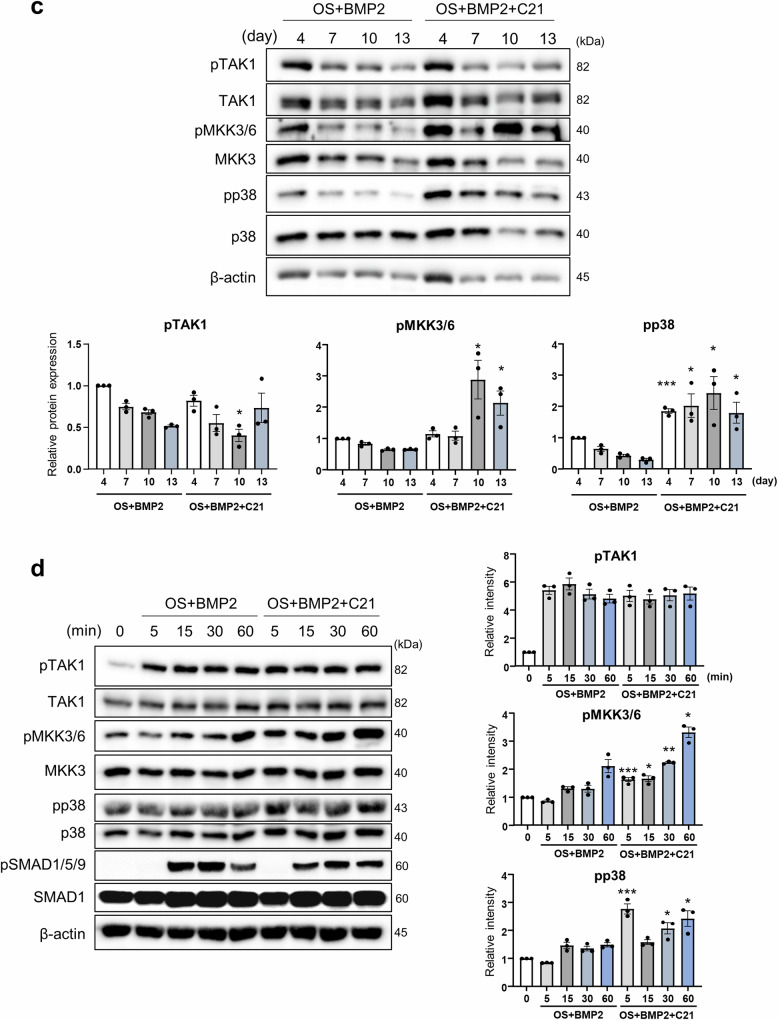
Analysis of the MKK3/6 and p38 signal transduction pathways affected by *Dock5* genetic deletion or chemical inhibition of DOCK5. **a** mBMSCs isolated from WT or *Dock5* KO mice stimulated with BMP2 in OS medium. Phosphorylation of the signaling molecules was analyzed at the indicated times (n = 2). **b** MC3T3-E1 cells induced with OS + BMP2 or OS + BMP2 + C21. Phosphorylation of the signaling molecules was analyzed at the indicated times (n = 3). **c** MC3T3-E1 cells were induced as described in **b**. Phosphorylation of the signaling molecules was analyzed at the indicated times (n = 3). **d** hBMSCs induced for osteoblast differentiation with OS + BMP2 or OS + BMP2 + C21. Phosphorylation of the signaling molecules was analyzed at the indicated times (n = 3). All relative intensity values were normalized to the total form. Statistical analysis was performed at each time point only, and comparisons at different time points were not conducted. *p < 0.05, **p < 0.01, and ***p < 0.001 as analyzed by two-tailed unpaired Student’s t test. TAK1 transforming growth factor-β-activated kinase 1, MKK3/6 mitogen-activated protein kinase kinase 3/6, SMAD1/5/9 mothers against decapentaplegic homolog 1/5/9, *Dock5* dedicator of cytokinesis 5, mBMSCs mouse bone marrow mesenchymal stem cells, WT wild-type, KO knockout, BMP2 bone morphogenetic protein 2, OS osteogenic induction, hBMSCs human bone marrow mesenchymal stem cells.

To understand how *Dock5* deletion or DOCK5 inhibition promotes osteoblast differentiation and bone formation in combination with BMP2, the effects on BMP2-mediated signaling pathways were examined in BMSCs and osteoblastic cells. First, BMSCs isolated from WT and *Dock5* KO mice were incubated with OS medium for 2 days, followed by treatment with BMP2 in OS medium for 0, 5, 15, 30, and 60 min. The phosphorylation of signaling molecules was examined. Specifically, phosphorylation of transforming growth factor-β-activated kinase 1 (TAK1) was increased by approximately 1.1-fold (13%) at 0 min. However, the activity of downstream molecules, such as mitogen-activated protein kinase kinase 3/6 (MKK3/6), was significantly greater in the BMSCs of *Dock5* KO mice than in those of WT mice, with approximately 9-, 13-, 5-, and 1.7-fold greater phosphorylation at 0, 5, 15, and 30 min, respectively, in response to BMP2 (Fig. 5a). Furthermore, BMP2 significantly increased the phosphorylation of p38 by 2.9-, 2.6-, 2-, and 2.4-fold at 0, 5, 15, and 30 min, respectively, in the BMSCs of *Dock5* KO mice compared with those of WT mice. The phosphorylation of other signaling molecules did not significantly differ between *Dock5* KO and WT BMSCs (Supplementary Fig. 4). Additionally, the phosphorylation of mothers against decapentaplegic homolog 1/5/9 (SMAD1/5/9) was increased, which may be associated with elevated levels of the SMAD1 protein induced by BMP2 (Fig. 5a), as described in a previous report^26^. These results demonstrate that the absence of *Dock5* could lead to the activation of MKK3/6 and p38 in response to BMP2.

To examine the effect of C21 on the BMP2-induced phosphorylation of signaling molecules, MC3T3-E1 cells were treated with BMP2 with or without C21. Compared with that of BMP2 alone, the BMP2-induced phosphorylation of TAK1 was not significantly increased by BMP2 + C21. However, the BMP2-induced phosphorylation of MKK3/6, phospho-mitogen-activated protein kinase kinase 1/2 (MEK1/2), extracellular signal-regulated kinase 1/2 (ERK1/2), and p38 was significantly increased by the addition of C21 from 5 to 60 min. No difference was observed in SMAD1/5/9 phosphorylation; however, protooncogene of *v-akt* (AKT; PKB) phosphorylation was significantly decreased from 15 to 60 min. Decreased levels of phosphorylation of c-JNK were also observed, albeit without statistical significance (Fig. 5b). In mBMSCs, the phosphorylation of MKK3/6 and p38 was also induced by C21 in the presence of BMP2 and OS (Supplementary Fig. 5). Interestingly, C21 tended to inhibit the phosphorylation of TAK1 in naïve MC3T3-E1 cells rather than in cells committed to the osteoblast lineage with OS medium (Supplementary Fig. 6, 7).

Furthermore, the effects of C21 on the BMP2-induced phosphorylation of TAK1, MKK3/6, and p38 in MC3T3-E1 cells undergoing osteoblast differentiation were examined for up to 13 days. MC3T3-E1 cells were treated with OS medium containing 20 ng/mL BMP2 and 50 μM C21 or vehicle for 4, 7, 10, or 13 days, and the phosphorylation of the signaling molecules was examined. Compared with that in control cells, TAK1 activity significantly decreased on Day 10 but slightly increased on Day 13 in experimental cells treated with C21. In addition, compared with that in control cells, MKK3/6 phosphorylation significantly increased from Days 10 to 13, and p38 activity increased continuously from Days 4 to 13. Therefore, C21 induced the activation of MKK3/6 and p38 more directly than did TAK1 (Fig. 5c). However, the phosphorylation of MEK1/2 and ERK1/2 did not differ according to C21 treatment during osteoblast differentiation (Supplementary Fig. 8).

To determine the presence of a similar signaling pathway in human cells, a phosphorylation assay was conducted on hBMSCs treated with OS medium containing 50 ng/mL BMP2 and either 25 μM C21 or DMSO. The results revealed no difference in the phosphorylation of TAK1, and a significant increase in the phosphorylation of MKK3/6 and p38 from 5 to 60 min was observed (Fig. 5d). These results strongly suggest that the inhibition of DOCK5 by C21 could activate TAK1 downstream molecules, including MKK3/6 and p38.

**Fig. 6:**
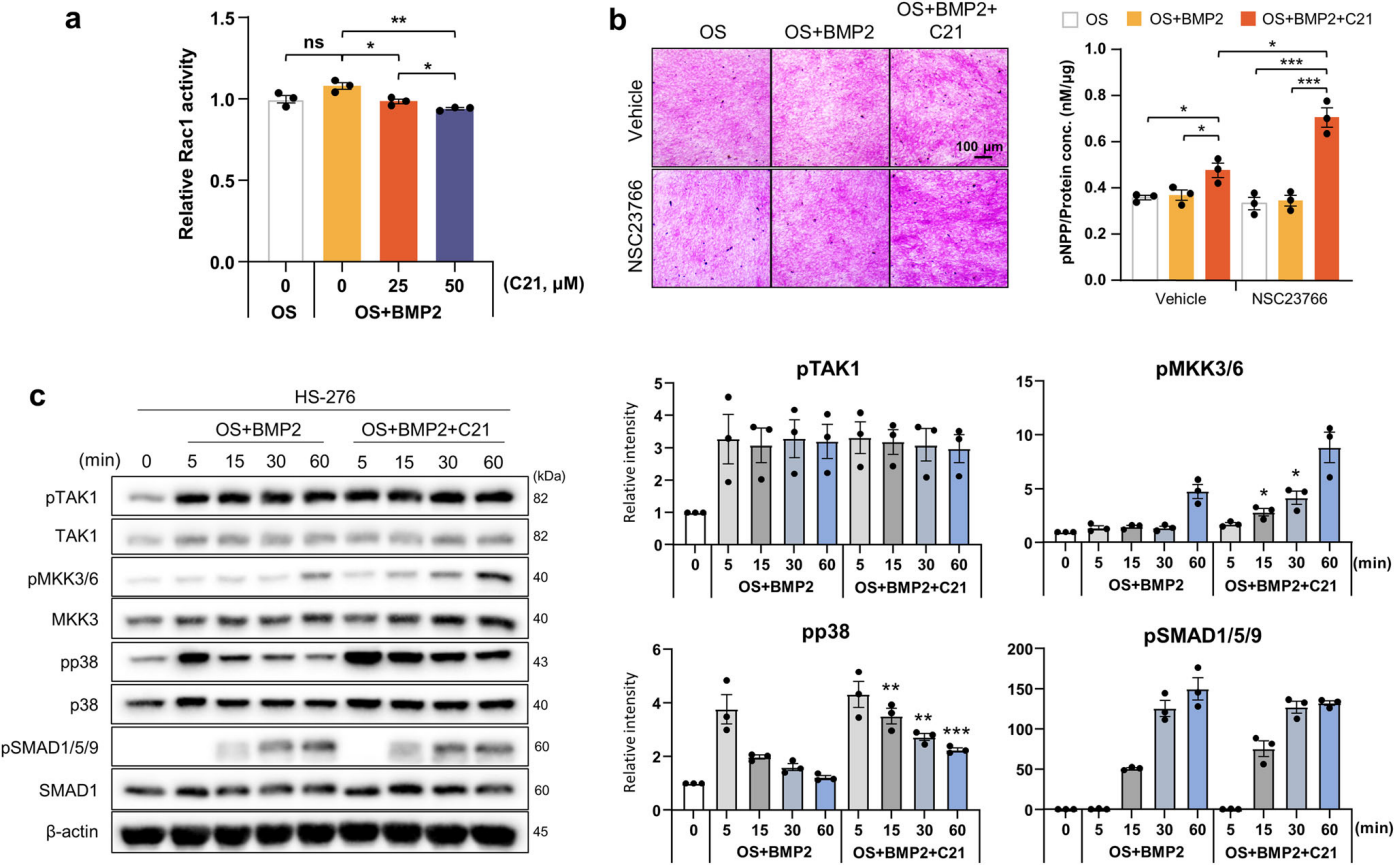
Analysis of TAK1 and Rac1 signal transduction pathways associated with the chemical inhibition of DOCK5. **a** Rac1 activity in MC3T3-E1 cells treated with BMP2 with or without C21. A dose-dependent inhibitory effect of C21 on Rac1 activity was observed (n = 3). **b** Effects of GEF inhibitors (C21 and NSC23766) on ALP activity and staining. MC3T3-E1 cells were treated as indicated, and ALP staining and activity assays were performed (n = 3). **c** Analysis of BMP2-induced phosphorylation in hBMSCs. hBMSCs were pretreated with 10 μM HS-276 (TAK1 inhibitor) for 24 h and stimulated with BMP2 in OS medium or with BMP2 + C21 in OS medium in the presence of HS-276 for the indicated times. The phosphorylation of signaling molecules was analyzed (n = 3). *p < 0.05, **p < 0.01, and ***p < 0.001 as analyzed by two-tailed unpaired Student’s t test (n = 3). TAK1: transforming growth factor-β-activated kinase 1, MKK3/6: mitogen-activated protein kinase kinase 3/6, SMAD1/5/9: mothers against decapentaplegic homolog 1/5/9, Rac1: ras-related C3 botulinum toxin substrate 1, BMP2: bone morphogenetic protein 2, ALP: alkaline phosphatase, GEF: guanine nucleotide exchange factor, hBMSCs: human bone marrow mesenchymal stem cells, OS: osteogenic induction.

To investigate whether DOCK5 regulates Rac1 activity in the modulation of MKK3/6 and p38, MC3T3-E1 cells were treated with BMP2 and the indicated concentrations of C21, and Rac1 activity was measured. Rac1 activity did not increase in response to BMP2. However, C21 inhibited Rac1 activity by 10% at 25 μM and by approximately 13% at 50 μM in the presence of BMP2 (Fig. 6a). Therefore, DOCK5 inhibition could suppress Rac1 activity. Previous studies have shown that Tiam1 can regulate Rac1 activity and thus affect osteoblast differentiation. In particular, NSC23766, a blocker of the Tiam1‒Rac1 interaction, was found to increase osteoblast differentiation by inhibiting Rac1 activity in C2C12 cells but had no effect on MC3T3‒E1 cells^11^. Therefore, we aimed to investigate whether Tiam1 also affects Rac1 activity and its relationship with DOCK5 in Rac1 activation. MC3T3-E1 cells were treated with or without NSC23766 or C21, and osteoblast differentiation was examined. Consistent with previous findings, no difference in ALP staining or activity was observed when the cells were treated with BMP2 alone or BMP2 + NSC23766 (Fig. 6b). However, when the cells were treated with C21 + BMP2, ALP staining and activity increased. Furthermore, ALP activity was enhanced by 1.5-fold when the cells were treated with both NSC23766 and C21 + BMP2 compared with C21 + BMP2 (Fig. 6b). These results suggest that, in the presence of BMP2, Rac1 activity could be modulated by DOCK5.

Conflicting results regarding mutual regulation between TAK1 and Rac1 have been reported. TAK1 is known to be regulated by Rac1 to induce nuclear factor kappa-light-chain-enhancer of activated B cells (NF-κB) activation and is necessary for the normal differentiation of osteoclast precursors^27^. However, TAK1-deficient cancer cells have increased surface expression of integrin α5β1 and active Rac1^28^. To examine the regulatory effects of TAK1 on Rac1, hBMSCs were stimulated with BMP2 in the presence or absence of HS-276 (a TAK1 inhibitor), as described in the Materials and Methods section. There was no difference in BMP2-induced TAK1 phosphorylation in the presence or absence of HS-276 (Supplementary Fig. 9). However, the phosphorylation of MKK3/6 and p38 by BMP2 was decreased in the presence of HS-276, indicating that BMP2-induced signaling pathways may involve TAK1 with MKK3/6 and p38 and that MKK3/6 and p38 may act downstream of TAK1 (Fig. 6c and Supplementary Fig. 9). Moreover, the phosphorylation of TAK1 was not modulated by C21; however, an increase in the phosphorylation of MKK3/6 and p38 was observed in response to the addition of C21 to BMP2 (Fig. 6c).

**Fig. 7:**
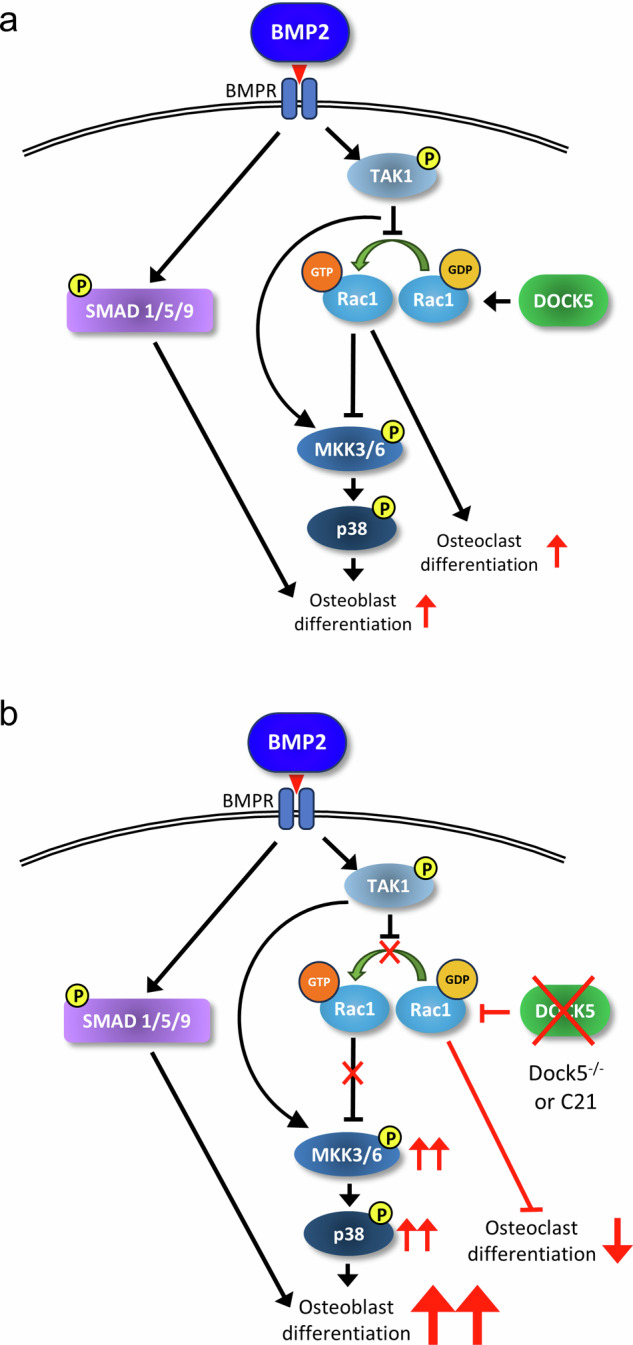
Signaling cascade of BMP2 in osteoblast differentiation. **a** Normal signaling cascade induced by BMP2. **b** Amplification of the BMP2-mediated MKK3/6 and p38 signaling pathways with DOCK5 inhibition or *Dock5* deletion. BMP2 bone morphogenetic protein 2, BMPR BMP receptor, TAK1 transforming growth factor-β-activated kinase 1, Rac1 ras-related C3 botulinum toxin substrate 1, DOCK5 dedicator of cytokinesis 5, GDP guanosine diphosphate, GTP guanosine triphosphate, SMAD1/5/9 mothers against decapentaplegic homolog 1/5/9, MKK3/6 mitogen-activated protein kinase kinase 3/6.

Collectively, the results demonstrated that the inhibition of DOCK5 could stimulate BMP2 signaling pathways involving MKK3/6 and p38 (Fig. 7). Therefore, osteoblast differentiation and bone regeneration induced by BMP2 may be synergistically enhanced through the inhibition of DOCK5 with the activation of the MKK3/6 and p38 signaling pathways.

## Discussion

In this study, we demonstrated that *Dock5* genetic deletion and chemical inhibition of DOCK5, a GEF for Rac1, enhanced the osteoblast differentiation of hBMSCs and mBMSCs and promoted bone regeneration by potentiating the BMP2-mediated MKK3/6 and p38 signaling pathways.

Several Rac1 GEFs have been demonstrated to regulate bone remodeling by acting on both osteoblasts and osteoclasts. ELMO1 is a Rac1 GEF, and its inhibition can suppress osteoclast function and bone loss; however, the effect of ELMO1 on osteoblasts has not been reported^29^. The in vivo findings differ somewhat from the in vitro findings. Transgenic mice in which Rac1 was deleted in preosteoblasts presented diminished bone formation, whereas the inhibition of Rac1 through the downregulation of triple functional domain (Trio) has been shown to promote osteoblast differentiation and inhibit the migration, invasion, and growth of osteosarcoma cells^30^. Additionally, knockdown of Tiam1 has been shown to increase BMP2-induced ALP activity^11^. Consistently, our results also demonstrated that chemical inhibition of DOCK5 and complete deletion of *Dock5* strongly enhanced the osteoblast differentiation of mBMSCs and hBMSCs in the presence of BMP2. These findings suggest a negative role of Rac1 GEFs in osteoblast differentiation in this context.

DOCK5 expression was previously reported to be very low in MSCs and MSC-derived osteoblasts^21^, and C21 did not alter osteoblast number or activity^31^. Nevertheless, our results showed that *Dock5* mRNA is detectable in mBMSCs and that osteogenic factors such as BMP2 can reduce Dock5 expression during osteoblast differentiation. In addition, mineral deposition and the expression of osteoblast differentiation marker genes in BMSCs undergoing osteoblast differentiation could be markedly enhanced by DOCK5 deficiency without the involvement of osteoclasts. This discrepancy may be attributed to differences in the experimental settings. Specifically, we used BMP2 to induce the osteoblast differentiation of BMSCs derived from *Dock5* KO mice, and there was a greater increase in mineral deposition and expression of osteoblast differentiation markers in *Dock5* KO mice than in WT mice. Consistently, the osteoblast differentiation of MC3T3-E1 cells was significantly increased by BMP2 in conjunction with C21. Therefore, these findings suggest that DOCK5 deficiency may strongly promote osteoblast differentiation and mineral deposition synergistically with BMP2.

Previously, a lack of DOCK5 was shown to have minimal effects on the differentiation of osteoclasts and affect the formation of the F-actin ring, inhibiting bone resorption^21^. However, we demonstrated that both osteoclast differentiation and bone-resorbing activity were significantly lower in *Dock5*-deficient BMMs than in WT BMMs. The location where a mutation is introduced, even if it disrupts the same gene, may be relevant. The *Dock5* KO mice used by Vives et al. to investigate bone resorption had a mutation in the DHR2 domain^32^; thus, the front part of DOCK5, including SH3 and DHR1, may still be produced in these mice. In contrast, the *Dock5* KO mice we generated in this study are complete KO mice because translation is terminated after exon 1, interrupting the SH3 domain. C21 also inhibited the osteoclast differentiation of WT BMMs as well as bone resorption. The differences in the differentiation and activity of osteoblasts and osteoclasts in different *Dock5* KO mice suggest diverse functional roles of DOCK5, and the exact mechanisms involved should be elucidated in the future.

As BMP2-mediated osteoblast differentiation was further promoted by the inhibition of the DOCK5 protein or complete deletion of *Dock5* in vitro, the role of *Dock5* in bone regeneration by BMP2 was analyzed in vivo. Currently, there are no animal models available for specifically evaluating the individual effects of osteoblasts. Therefore, bone regeneration was assessed in three mouse models: calvarial defects, cranial ectopic bone formation, and subcutaneous ectopic bone formation. Typical bone regeneration was evaluated in the calvarial defect model using *Dock5* KO and WT mice, and bone regeneration in *Dock5* KO mice with calvarial defects was markedly enhanced, as demonstrated by micro-CT and histology. Inhibition of osteoclasts could stimulate osteoblast differentiation, and *Dock5* could regulate both osteoclast differentiation (observed in this study) and function as reported previously^21,25^; thus, the possibility of low osteoclast activity caused by *Dock5* deletion could not be excluded in enhanced bone regeneration in *Dock5* KO mice^21^. Therefore, we investigated whether *Dock5* deletion in periosteal MSCs, which can be a cellular source for fracture healing and regeneration in long bones^33^, stimulates BMP2-induced bone formation^34,35^. Although *Dock5* HE and WT mice exhibited similarly low levels of bone formation, *Dock5* KO mice presented high BV, Tb.Th, and bone maturity in the presence of BMP2. These results suggest that the periosteal MSCs may have greater potential to differentiate into osteoblasts and thus increase the bone mass in *Dock5* KO mice than in WT mice. We also investigated whether bone formation could be synergistically induced by DOCK5 inhibition and BMP2 in a more stringent environment, such as a subcutaneous site. Consistent with the in vitro results, BMP2 and C21 significantly enhanced ectopic bone formation and bone maturation. A previous study showed that daily injection of C21 could inhibit osteoclast function and thus prevent ovariectomy-induced bone loss^31^. However, in our experimental setting, soaking a collagen sponge in C21 and BMP2 once for implantation was sufficient to synergistically induce ectopic bone formation. These results strongly suggest that DOCK5 inhibition and *Dock5* deletion in MSCs stimulate BMP2-induced bone regeneration and formation in vivo.

Understanding how DOCK5 inhibition potentiates BMP2-induced osteoblast differentiation and bone regeneration is important. BMP2 is one of the most potent osteogenic factors and is widely used in clinical settings for bone regeneration. BMP2 can activate multiple signaling pathways depending on microenvironmental conditions, sometimes leading to adverse events^4^. In particular, BMP2 can induce the activation of SMAD-dependent and non-SMAD-dependent pathways, including the TAK1, MKK3/6, AKT, JNK, and p38 pathways^7–9,11,12,26,36^.

TAK1 has been reported to regulate Rac1^28^; thus, it can be hypothesized that TAK1 may regulate the activity of Rac1 through DOCK5. Our results demonstrated that BMP2-induced TAK1 phosphorylation was not affected by C21 in human or mouse cells. In addition, TAK1 inhibition by HS-276 did not modulate TAK1 phosphorylation in response to BMP2. These results suggest that TAK1 may function upstream of DOCK5-Rac1 in BMP2 signaling. Interestingly, C21 tended to inhibit the phosphorylation of TAK1 in naïve MC3T3-E1 cells rather than in cells committed to the osteoblast lineage with OS medium. These results imply that TAK1 phosphorylation could be differentially regulated depending on the commitment stage of the cells and that TAK1 phosphorylation may be regulated by DOCK5 only in naïve cells but not in osteoblast-committed cells. Considering that the activation of TAK1 could lead to the induction of inflammatory responses in normal cells^37^, the results revealed a positive effect of DOCK5 inhibition on the regulation of inflammation. Specifically, DOCK5 inhibition might prevent BMP2-mediated inflammation by alleviating TAK1 activation. Although TAK1 inhibition via HS-276 also markedly suppressed the BMP2-induced phosphorylation of MKK3/6 and p38, C21 significantly reversed their phosphorylation. Therefore, it is reasonable to locate DOCK5 between TAK1 and MKK3/6 and p38 in BMP2 signaling pathways.

In summary, DOCK5 may play a crucial role in regulating Rac1 activity in the presence of BMP2 downstream of TAK1. The inhibition of DOCK5 could suppress osteoclast differentiation while promoting osteoblast differentiation. BMP2-mediated osteoblast differentiation may occur primarily through the TAK1-MKK3/6-p38 signaling pathway; however, DOCK5 may function via an alternate route and act on MKK3/6 and p38 in osteoblast differentiation. When BMP2 and DOCK5 are combined, there may be a synergistic increase in osteogenic differentiation, as demonstrated by both in vitro and in vivo findings. Therefore, inhibition of DOCK5 with BMP2 treatment could be a promising strategy for enhancing osteoblast differentiation and bone regeneration while controlling for negative factors such as osteoclast differentiation.

## Supplementary information

Supplementary Information
