# Supplementary material for: The guanine nucleotide exchange factor DOCK5 negatively regulates osteoblast differentiation and BMP2-induced bone regeneration via the MKK3/6 and p38 signaling pathways
Source: Exp Mol Med. 2025 Jan 1;57(1):86–103. doi: 10.1038/s12276-024-01372-2 (PMC11799167; doi:10.1038/s12276-024-01372-2)
Supplement: Supplementary file 1 — Supplementary Information [file 12276_2024_1372_MOESM1_ESM.pdf]

Supplementary Information

The guanine nucleotide exchange factor DOCK5 negatively regulates osteoblast differentiation and BMP2-induced bone regeneration via the MKK3/6 and p38 signaling pathways

Authors: Ju Ang Kim[1], Soomin Im[1], Jiwon Lim[1], Jung Min Hong[1], Hye Jung Ihn[2], Jong-Sup Bae[3], Jung-Eun Kim[4], Yong Chul Bae[5] and Eui Kyun Park[1*]

Affiliations: [1]Department of Pathology and Regenerative Medicine, School of Dentistry, IHBR, Kyungpook National University, Daegu 41940, Republic of Korea. [2]Cell and Matrix Research Institute, Kyungpook National University, Daegu 41944, Republic of Korea. [3]Research Institute of Pharmaceutical Sciences, College of Pharmacy, Kyungpook National University, Daegu 41566, Republic of Korea. [4]Department of Molecular Medicine, Cell and Matrix Research Institute, School of Medicine, Kyungpook National University, Daegu 41944, Republic of Korea. [5]Department of Anatomy and Neurobiology, School of Dentistry, Kyungpook National University, Daegu 41940, Republic of Korea. (* Correspondence)

Supplementary Materials and Methods

Cell viability assay

The cytocompatibility of C21 was assessed by MTT assay. MC3T3-E1 cells (7,000 cells) or hBMSCs (700 cells) were seeded into 96-well plates (n = 3). A day after seeding, various concentrations of C21 were added for 1, 3, and 6 days. At the indicated time, MTT reagent (5 μg/mL) was added for 2 h at 37℃. Formazan was solubilized with DMSO. The absorbance of solubilized formazan was measured at 570 nm using an Epoch 96-well microplate reader (BioTek Instruments).

Osteoclast differentiation and TRAP staining

Bone marrow macrophages (BMMs) were isolated from mouse femur and tibial bones as previously described[1]. The cells were cultured in α-MEM containing 10% FBS on a Petri dish. After 24 h, non-adherent cells were separated using a Histopaque density gradient medium (Sigma-Aldrich; Merck KGaA, St. Louis, MO, USA) following a 3-day culture in the presence of M-CSF (30 ng/mL) to obtain BMMs. For osteoclast differentiation, BMMs were cultured with RANKL (20 ng/mL) and M-CSF (10 ng/mL) in 96-well plates. The effect of C21 on osteoclast differentiation was investigated by culturing BMMs with RANKL and M-CSF in the presence of varying C21 concentrations (0, 25, and 50 μM). Five days post-RANKL treatment, the cells were fixed in 4% paraformaldehyde for 15 min at room temperature and stained using a TRAP staining kit following the manufacturer's instructions (Cosmo Bio Co). TRAP-positive multinucleated cells with three or more nuclei were identified and counted as osteoclasts.

Immunofluorescence staining and bone resorption pit assay

BMMs were plated on glass coverslips and cultured with RANKL (20 ng/mL) and M-CSF (10 ng/mL) for 5 days. The cells were then fixed with 4% paraformaldehyde for 15 min at room temperature, and immunofluorescence staining was performed with Nfatc1 primary antibody (1:500, sc-7294; Santa Cruz Biotechnology) and Alexa Fluor 488-conjugated secondary antibody (1:100, A-11059; Thermo Fisher Scientific) as described previously[2]. Staining with rhodamine-conjugated phalloidin (100 nM, PHDR1; Cytoskeleton, Inc.) and 4’,6-diamidino-2-phenylindole dihydrochloride (DAPI, 1:10,000; Santa Cruz Biotechnology) was performed for 1 h at room temperature. Fluorescence images were captured using a Leica DM2500 microscope with Leica Application Suite X (LAS X, v3.00.15697; Leica Microsystems GmbH). Image alignment was performed using ImageJ software (version 1.52a; National Institutes of Health).

For resorption pit assay, 2.5 × 10^4 BMMs were seeded on bone slices (IDS Nordic) and incubated with RANKL (20 ng/mL) and M-CSF (10 ng/mL) for 5 days at 37°C. The bone slices were then washed and ~~briefly soaked in hematoxylin solution for 30 s at room temperature~~ to visualize the resorption pits. The resorbed areas were observed under a microscope and captured using Leica Application Suite X (LAS, v4.12.0). The pit area was quantified using ImageJ software.

Reverse transcription-quantitative PCR (*RT-qPCR)*

To analyze the mRNA expression of specific osteogenic marker genes, the cells were collected on the first day (OS day 4) and third day (OS day 7) after treatment with C21 and rhBMP2, washed twice with ice-cold PBS, and lysed with 200 µL/cm² of Tri-solution™ (Bio Science Technology). Total RNA was extracted following the manufacturer's instructions, and 1 µg of RNA was used for cDNA synthesis with Superscript II (Enzynomics), as described in the manufacturer's manual (Eppendorf). For quantitative PCR, 2 µL of cDNA was used in a 20 µL PCR mixture containing specific primer sets (Supplementary Table 1) and 2X SYBR master mix (Applied Biosystems). The PCR protocol involved a hold stage (50℃ for 2 min, 95℃ for 10 min), 45 cycles of PCR stage (95℃ for 15 s, 58~62℃ for 30 s, 72℃ for 30 s), and a melt curve stage (95℃ for 15 s, 60℃ for 1 min, 95℃ for 15 s) performed using a Real-time PCR machine (Thermo Fisher Scientific). The data were analyzed using the QuantStudio™ Design and Analysis software (v1.5.2). Human or mouse glyceraldehyde-3-phosphate dehydrogenase (GAPDH) was used as the internal control. Relative gene expression data were quantified using the $2^{-\Delta\Delta Cq}$ method.

Condition for micro-CT analysis

The scanning parameters were set at 70 kV and 142 µA with an Al 0.5 mm filter and an exposure time of 658 ms. The resolution was maintained at 10 µm per voxel with a 2452 × 1640 pixel image matrix. To derive 2D images from the reconstructed sections, DataViewer software (Bruker-micro-CT, ver. 1.5.6.2) was used. Measurements of the sagittal suture area were conducted on digitally recorded projections of micro-CT 3D images using the CTAn image analysis software (Bruker-micro-CT, ver. 1.19.4.0+).

Immunohistochemistry

Decalcified bone was cryosectioned with a thickness of 6 µm. The sections were treated twice with 8% SDS in PBS and twice with 25% amino alcohol for 10 min each at room temperature. The sections were blocked with 5% normal goat serum and 1% BSA in 0.25% Triton X-100/PBS for 1 h at room temperature. For cathepsin K staining, incubation with anti-cathepsin K (1:50; Santa Cruz Biotechnology) was performed overnight at 4°C, followed by treatment with Alexa-594 conjugated secondary antibody (1:400, A11012; Invitrogen) and DAPI (1:10,000) for 1 h at room temperature. For osteocalcin (OC) staining, anti-OC primary antibody (1:100, ab93876; Abcam), Alexa-488 conjugated secondary antibody (1:500, R37116; Invitrogen), and DAPI (1:10,000) were used. Fluorescence images were captured as described above, and the intensity of the signals was analyzed using ImageJ software.

Supplementary Figures

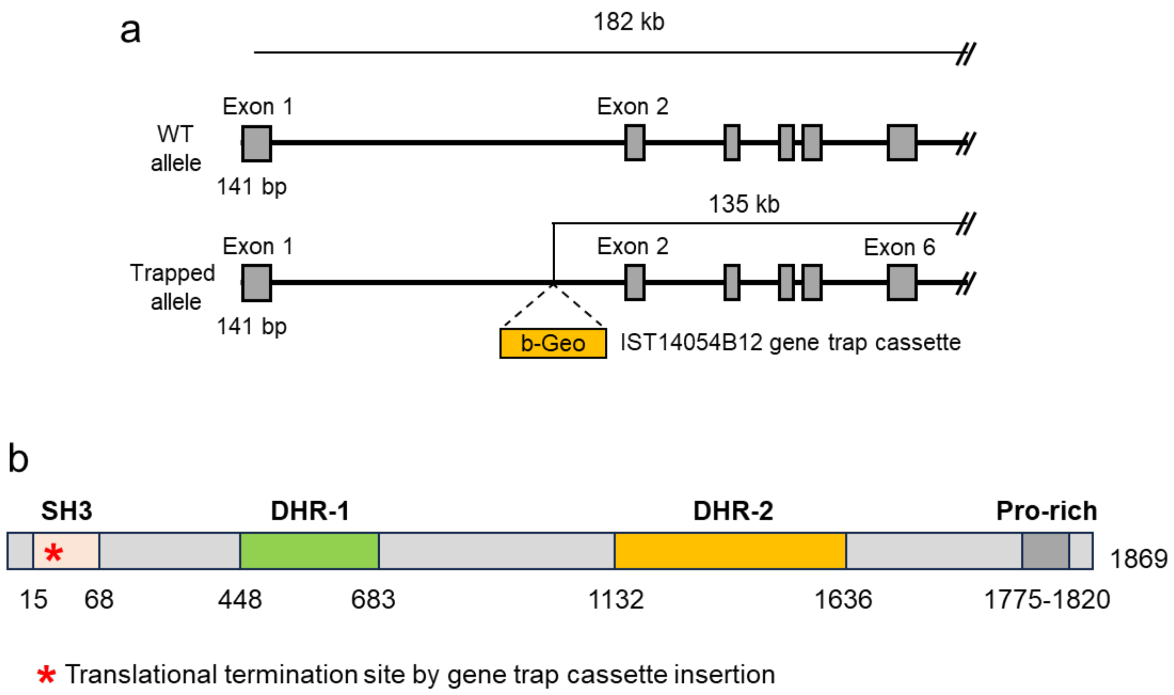

Supplementary Fig. 1 Generation of complete *Dock5* KO mice. **a** WT allele of *Dock5* and trapped allele with b-Geo cassette (beta-Geo) for generating *Dock5* KO mice. **b** Domain structure of DOCK5 protein. The translational termination site is marked with a red asterisk (*).

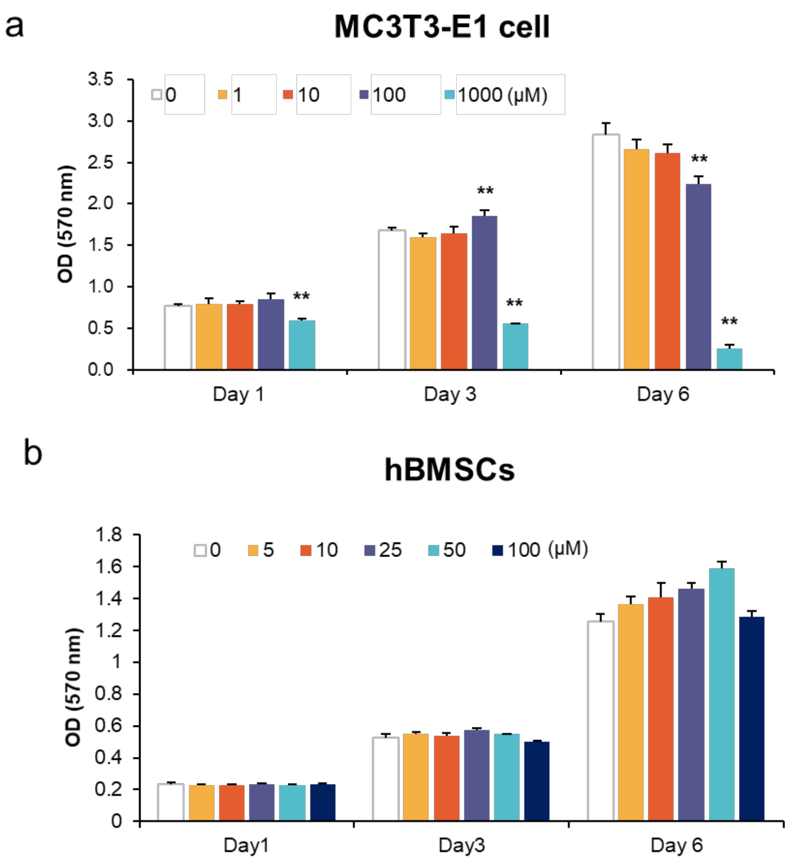

Supplementary Fig. 2 MTT assay of cell viability and proliferation. **a** MC3T3-E1 cell viability screened using 0, 1, 10, 100, and 1000 μM C21 on day 1, 3, and 6 (n = 3). **b** C21 concentration in hBMSCs determined using 0, 5, 10, 25, 50, and 100 μM C21 on day 1, 3, and 6 (n = 3).

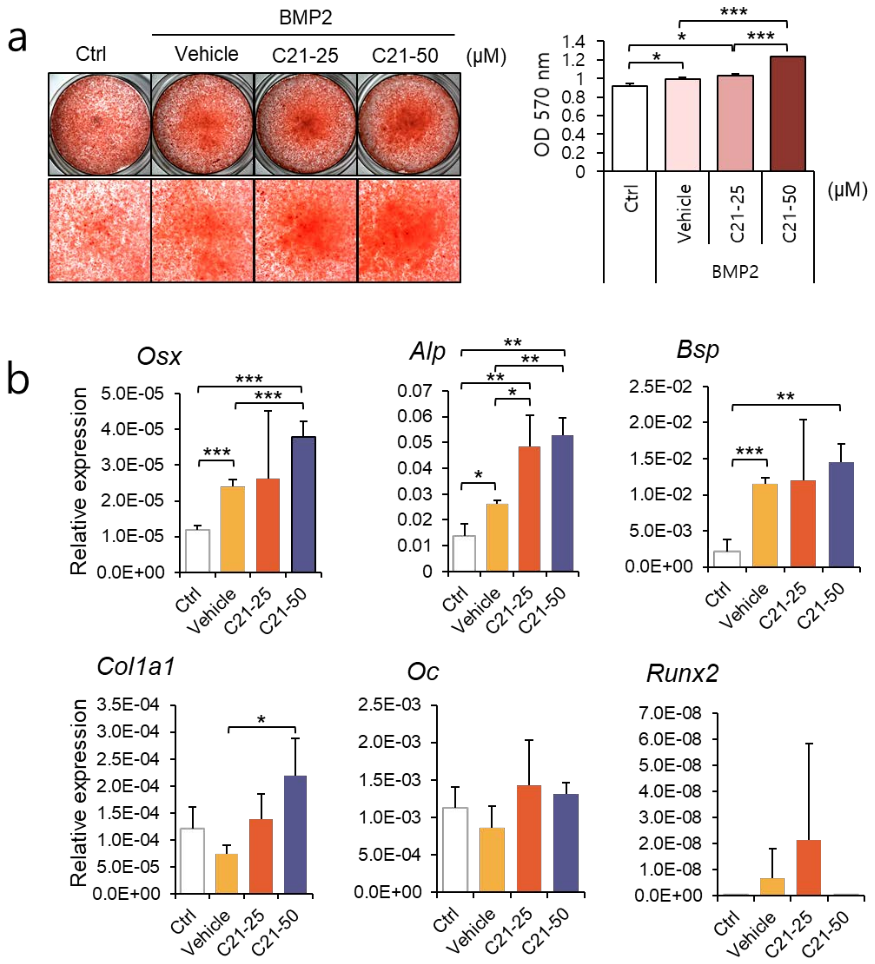

Supplementary Fig. 3 Effect of C21 on osteoblast differentiation of mBMSCs. **a** mBMSCs were treated with rhBMP2 and 25 or 50 μM C21 for 8 days. Mineral deposition was visualized with Alizarin Red S solution (n = 3). **b** RT-qPCR for the expression of osteogenesis-specific marker genes in cells undergoing osteoblast differentiation on day 3 (n = 3). The expression of each gene was normalized to *Gapdh* expression. *p < 0.05, **p < 0.01, and ***p < 0.001 as analyzed by one-way ANOVA with Tukey's multiple comparison post-hoc test.

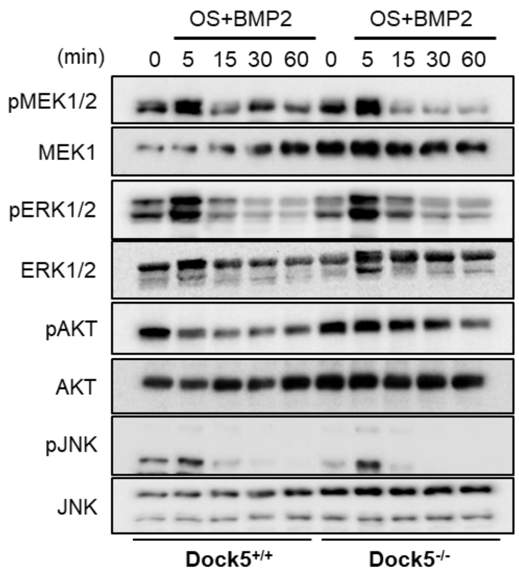

Supplementary Fig. 4 Phosphorylation of signaling molecules in WT and *Dock5* KO BMSCs in response to OS + BMP2. mBMSCs isolated from WT or *Dock5* KO mice were stimulated with BMP2 in OS medium, and the phosphorylation of signaling molecules was analyzed at the indicated time points.

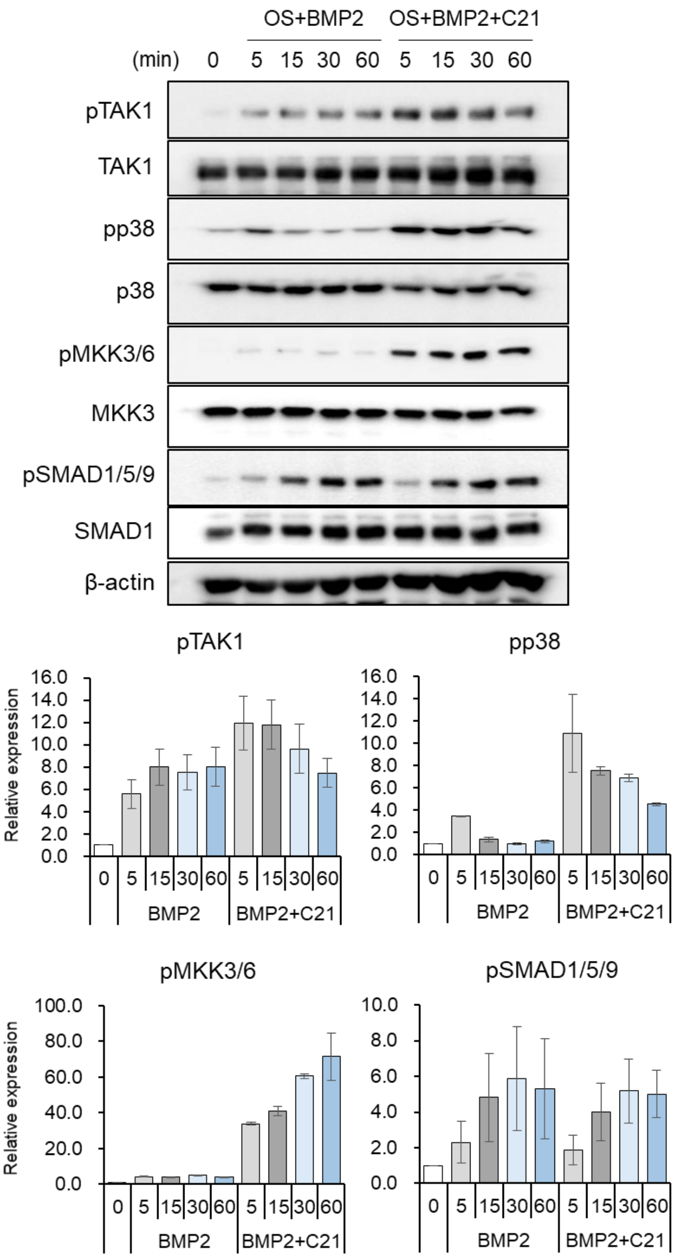

Supplementary Fig. 5 Phosphorylation of signaling molecules in mBMSCs in response to OS + BMP2 or OS + BMP2 + C21. All relative intensity values were normalized to the total form.

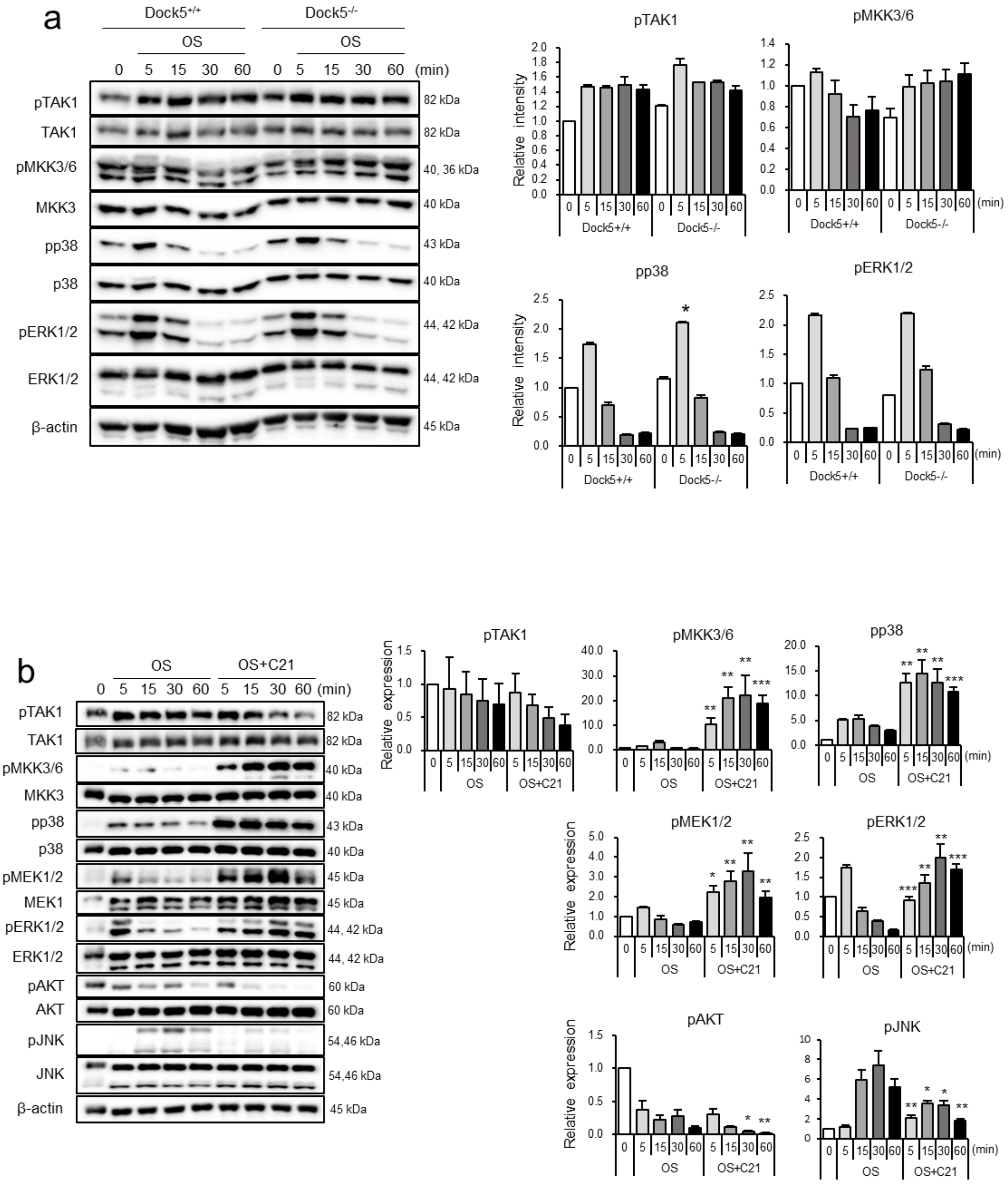

Supplementary Fig. 6 Phosphorylation of signaling molecules under OS. **a** Phosphorylation of signaling molecules in mBMSCs. The cells were isolated from *Dock5* WT and KO mice and treated with OS medium. The intensity of phosphorylated signaling molecules was normalized to their total form and β-actin. The level of phosphorylation was quantified (n = 2). **b** Phosphorylation of signaling molecules induced by OS medium in the presence or absence of C21 (100 μM). Western blotting was used to visualize and quantify the phosphorylation of signaling molecules compared to the total form (n = 3).

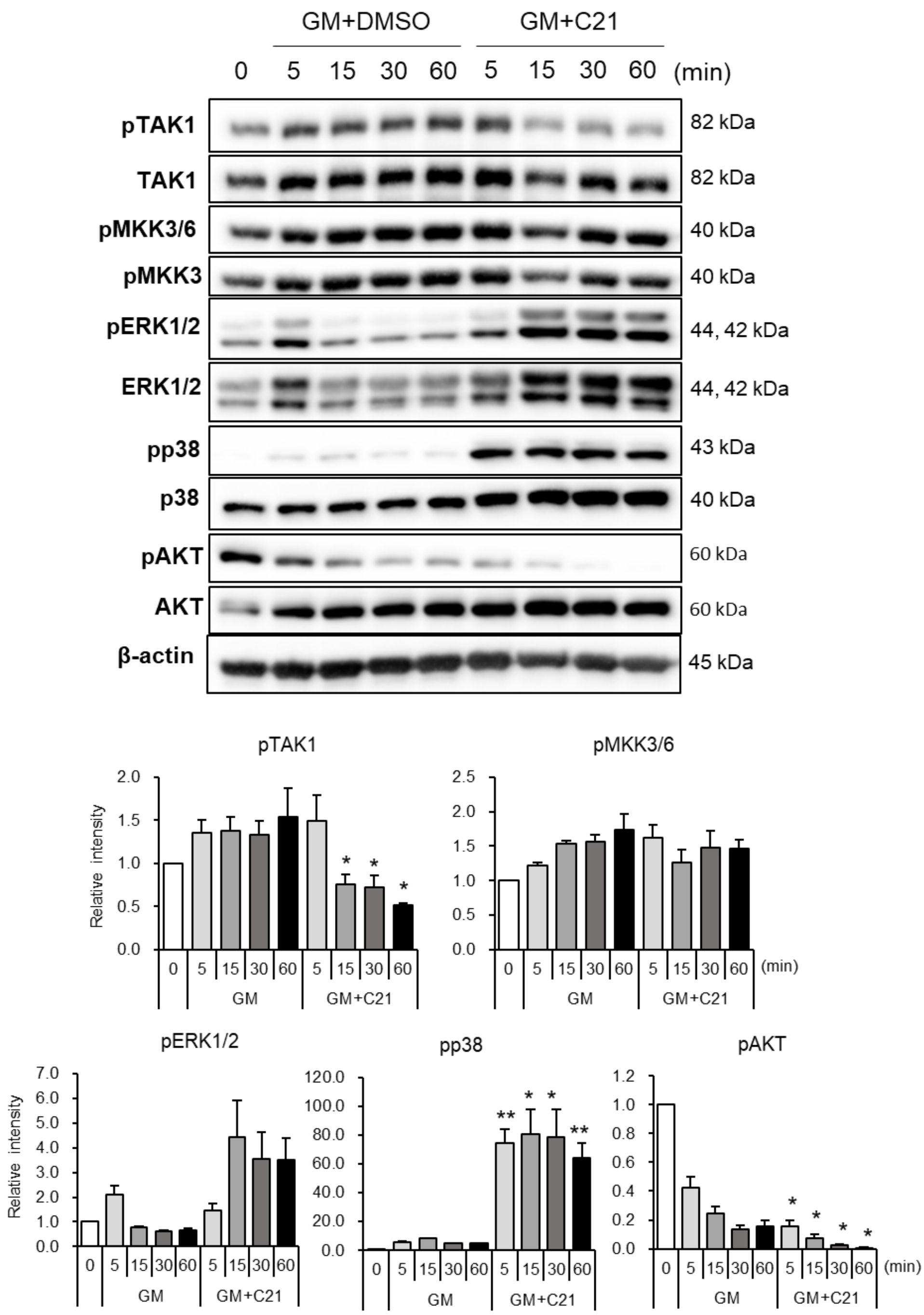

Supplementary Fig. 7 Western blotting showing the effects of C21 on the phosphorylation of signaling molecules under normal growth medium (GM) conditions in MC3T3-E1 cells. The cells were treated with DMSO or 100 μM C21 in α-MEM containing 0.3% FBS for the indicated times. The phosphorylation level of each protein was normalized to its total form. Statistical significance was analyzed at the same time point. **p* < 0.05 and ***p* < 0.01 as analyzed by two-tailed unpaired Student's t-test (n = 3).

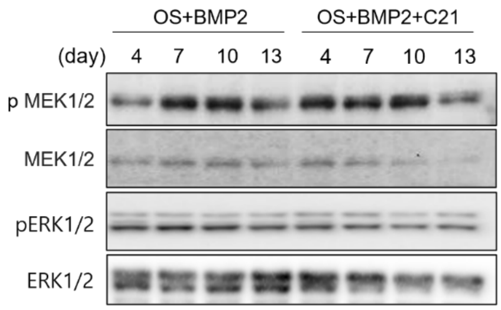

Supplementary Fig. 8 Phosphorylation of the signaling molecules MEK1/2 and ERK1/2 in MC3T3-E1 cells undergoing osteoblast differentiation was analyzed at the indicated days.

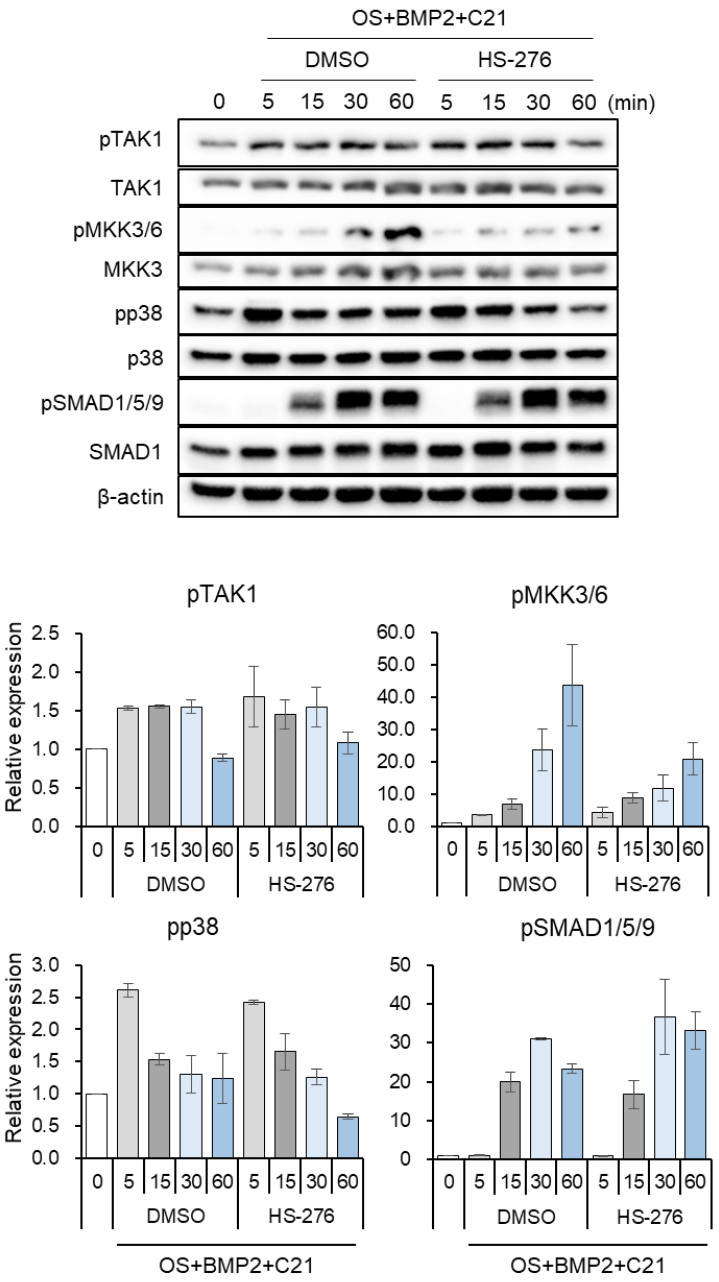

Supplementary Fig. 9 Analysis of BMP2-induced phosphorylation in hBMSCs. hBMSCs were pre-treated with DMSO or 10 μM HS-276 (TAK1 inhibitor) for 24 h and stimulated with BMP2 + C21 in OS medium in the presence of DMSO or HS-276 for the indicated times. Phosphorylation of the signaling molecules was analyzed (n = 2).

Supplementary Table 1. Primer sequence for RT-qPCR (F: forward, R: reverse)

Gene		Sequence (5' → 3')	TM (℃)	Amlicon size (bp)	RefSeq NCBI no.
mGapdh	F	ATGACATCAAGAAGGTGGTG	55	177	NM_001411843.1
	R	CATACCAGGAAATGAGCTTG			
mMT1-MMP	F	AGTCAGGGTCACCCACAAAGA	60	74	NM_008608.4
	R	TTTGGGCTTATCTGGGACAGA			
mDock5	F	GGGCCTAGGCAGTCTCTTGA	60	111	NM_177780.3
	R	GGGAAGCCCTGTCCGTAGTAT			
mNfatc1	F	ACCACCTTTCCGCAACCA	60	72	NM_001164112.1
	R	TTCCGTTTCCCGTTGCA			
mTrap	F	TCCCCAATGCCCCATTC	59	63	NM_001102405.1
	R	CGGTTCTGGCGATCTCTTTG			
mCtsk	F	GGCTCTGGAGGCGGCTAT	61	66	NM_007802.4
	R	AGAGTCAATGCCTCCGTTCTG			
mRunx2	F	CCTGAACTCTGCACCAAGTCCT	62	125	NM_009820.6
	R	TCATCTGGCTCAGATAGGAAGGG			
mBsp	F	AATGGAGACGGCGATAGTTCCG	62	126	NM_008318.3
	R	GGAAAGTGTGGAGTTCTCTGCC			
mOcn	F	GCAATAAGGTAGTGAACAGAC	59	157	NM_007541.3
	R	CCATAGATGCGTTTGTAGGCG			
mOsx	F	GGCTTTTCTGCGGCAAGAGGTT	55	139	NM_001348205.1
	R	CGCTGATGTTTGCTCAAGTGGTC			
mCol1a1	F	CCTAATGCTGCCTTTTCTGC	58	199	NM_007742.4
	R	ATGTCCCAGCAGGATTTGAG			
mAlp	F	AACCCAGACACAAGCATTCC	60	151	NM_007431.3
	R	GAGAGCCAAGGGTCAGTCAG			
hCOL1A1	F	GATTCCCTGGACCTAAAGGTGC	64	107	NM_000088.4
	R	AGCCTCTCCATCTTTGCCAGCA			
hRUNX2	F	CCCAGTATGAGAGTAGGTGTCC	64	149	NM_001015051.4
	R	GGGTAAGACTGGTCATAGGACC			
hBSP	F	GGCAGTAGTGACTCATCCGAAG	62	140	NM_004967.4
	R	GAAAGTGTGGTATTCTCAGCCTC			
hOC	F	CGCTACCTGTATCAATGGCTGG	63	123	NM_199173.6
	R	CTCCTGAAAGCCGATGTGGTCA			
hOSX	F	TTCTGCGGCAAGAGGTTCACTC	63	129	NM_001300837.2
	R	GTGTTTGCTCAGGTGGTCGCTT			
hALP	F	CAACGAGGTCATCTCCGTGATG	63	129	NM_001632.5
	R	TACCAGTTGCGGTTCACCGTGT			
hGAPDH	F	ATGGGGAAGGTGAAGGTCG	58	108	NM_001357943.2
	R	GGGGTCATTGATGGCAACAATA			

References

1 Kim, J. A. *et al.* Britanin inhibits titanium wear particle-induced osteolysis and osteoclastogenesis. *Mol. Med. Rep.* **28**, 205 (2023).

2 Lim, S. *et al.* Suppressive effects of (-)-tubaic acid on RANKL-induced osteoclast differentiation and bone resorption. *Anim. Cells Syst. (Seoul)* **27**, 1-9 (2023).
